# Volumetric chemical imaging by stimulated Raman projection microscopy and tomography

**DOI:** 10.1038/ncomms15117

**Published:** 2017-04-24

**Authors:** Xueli Chen, Chi Zhang, Peng Lin, Kai-Chih Huang, Jimin Liang, Jie Tian, Ji-Xin Cheng

**Affiliations:** 1 Engineering Research Center of Molecular and Neuro Imaging of Ministry of Education & School of Life Science and Technology, Xidian University, Xi'an, Shaanxi 710126, China; 2 Weldon School of Biomedical Engineering, Purdue University, West Lafayette, Indiana 47907, USA; 3 Institute of Automation, Chinese Academy of Science, Beijing 100190, China; 4 Department of Chemistry, Purdue University, West Lafayette, Indiana 47907, USA

## Abstract

Volumetric imaging allows global understanding of three-dimensional (3D) complex systems. Light-sheet fluorescence microscopy and optical projection tomography have been reported to image 3D volumes with high resolutions and at high speeds. Such methods, however, usually rely on fluorescent labels for chemical targeting, which could perturb the biological functionality in living systems. We demonstrate Bessel-beam-based stimulated Raman projection (SRP) microscopy and tomography for label-free volumetric chemical imaging. Our SRP microscope enables fast quantitation of chemicals in a 3D volume through a two-dimensional lateral scan. Furthermore, combining SRP and sample rotation, we demonstrate the SRP tomography that can reconstruct the 3D distribution of chemical compositions with optical spatial resolution at a higher speed than the Gaussian-beam-based stimulated Raman scattering sectioning imaging can. We explore the potential of our SRP technology by mapping polymer particles in 3D volumes and lipid droplets in adipose cells.

## Abstract (web summary)

Recent advances have enabled high-speed three-dimensional optical imaging through the use of fluorescent markers. Here, Chen *et al*. integrate stimulated Raman imaging into those methods, enabling the label-free and chemically specific volumetric imaging of complex samples.

Volumetric imaging enables quantitative and global measurements of a three-dimensional (3D) complex system. It allowed quantitation of molecules in the whole volume of a specimen, and has proven to be invaluable in the studies of cell metabolism, brain function, and developmental biology^1^,^2^,^3^,^4^. The simplest way to image a volume is through optical sectioning in the axial direction. In such a scheme, the laser scans in the lateral plane to collect a two-dimensional (2D) image, and then the laser focus moves along the axial direction to acquire an image stack for 3D reconstruction. Such a method requires tightly focused laser beams and good axial sectioning capability. Confocal or multiphoton fluorescence microscopy can section a volume with good axial resolution^5^,^6^. Coherent Raman scattering microscopy, having a similar sectioning ability, can map the chemical compositions in a volume based on molecular vibrations^7^,^8^. Such a sectioning approach, however, can be time-consuming for a volume hundreds of micrometres across^9^.

Light-sheet microscopy overcame this limitation by scanning an axially elongated laser beam to cover the completely 3D volume in a single 2D lateral scan. For example, the light-sheet fluorescence microscopy has achieved high-resolution and high-speed volumetric imaging of biological samples ranging from single cells to whole embryos^10^,^11^. Despite its wide applications^12^,^13^,^14^,^15^, the fluorescent labels used in light-sheet fluorescence microscopy might cause strong perturbation to biological functionalities in living systems, especially when labelling small metabolic molecules. Fluorescent labels can also induce other issues, such as non-specific targeting and photobleaching^16^. Light-sheet Raman microscopy has been reported to enable label-free volumetric imaging of chemicals in a volume^17^,^18^,^19^,^20^,^21^. However, the spontaneous Raman scattering suffers from low signal level and strong autofluorescence background, especially in living samples^19^. Furthermore, the image quality in the light-sheet microscopy often degenerates as the distance from the sample surface to the objective increases^22^.

Another approach for volumetric imaging is through tomography, in which images were collected from many different angles around the sample. The 3D information can be then reconstructed using the angle-dependent images. Tomography can overcome the depth-induced deterioration on image quality in light-sheet techniques^22^. On the basis of the light transmission or emission, optical projection tomography (OPT) can produce isotropic, high-resolution image of a specimen in 3D (refs 2, 9, 23, 24). Nonetheless, the transmission OPT does not have chemical contrast, whereas the fluorescence emission OPT subjects to the same issues in fluorescence microscopy for the quantification of metabolic molecules in living samples. Spontaneous Raman tomography was reported to image the chemical compositions of specimens in 3D (refs 25, 26, 27), yet it has low image acquisition speed due to the inherently low signal level of spontaneous Raman scattering. Furthermore, the spontaneous Raman tomography is based on the collection of diffused photons, leaving the technique with low spatial resolution, usually on the scale of millimetres. Such a low spatial resolution was caused by the physical nature of the diffuse light propagation in turbid medium^28^,^29^.

Here we demonstrate the first Bessel-beam-based stimulated Raman projection (SRP) microscopy and tomography for high-speed volumetric chemical imaging. Our SRP method is built upon the concept that the detected signal is an integration of Stimulated Raman Scattering (SRS) intensity along the Rayleigh length of the input Bessel beams, which remain their focuses along axial direction for a long distance. Compared to the Gaussian beam SRS microscopy, the Bessel beam SRP microscopy is able to quantify the total chemical compositions of a 3D volume in a 2D lateral scan. By rotating the sample, the 3D chemical distribution inside the sample volume can be reconstructed by the SRP tomography with isotropic spatial resolution. We first theoretically simulate the generation of SRP signal using Bessel pump and Stokes beams. The experimental characterization results of the system show good agreement with our simulation results. Using polymer particles and single adipose cells, we further exemplify the potential of our SRP technology for *in vivo* volumetric chemical imaging of live samples. Our results collectively prove that the SRP microscopy and tomography allow fast quantitation of chemical compositions in a 3D volume.

## Results

### Volumetric imaging based on stimulated Raman scattering

**Figure 1:**
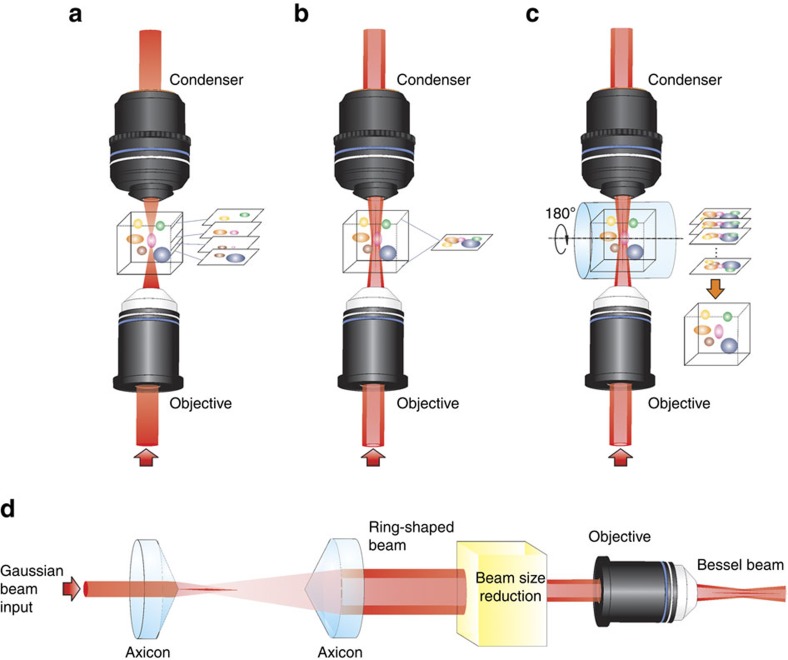
Volumetric imaging modalities based on stimulated Raman scattering. (**a**) Sectional imaging by the conventional Gaussian beam stimulated Raman scattering microscopy. (**b**) Stimulated Raman projection (SRP) microscopic imaging based on Bessel beams. (**c**) SRP tomographic imaging based on Bessel beams. (**d**) The generation of a Bessel beam using a pair of axicons and an objective lens.

Conventionally, the SRS imaging relies on 2D lateral scanning of tightly focused Gaussian laser beams. To image through a 3D volume, either the laser focus or the sample needs to be swept along the axial direction at one image per step. The image stack obtained from such a scheme can be used to reconstruct the 3D volume, as shown in Fig. 1a. To quantify the total chemical compositions in the volume, such a sectioning imaging scheme can be time-consuming, especially for a large volume that requires many image slices to reconstruct. Bessel beam can remain tightly focused for a long distance. Using Bessel beams for excitation, the SRS signal can be integrated along the axial direction of the sample. Scanning the Bessel excitation beams two-dimensionally on the lateral plane can therefore generate a projection image containing the total amount of chemical compositions in a volume. Such an imaging modality, termed SRP microscopy in this paper, is illustrated in Fig. 1b. Though SRP microscopy allows for high-speed quantitation of total chemical compositions in a volume, it loses axial resolution. To reconstruct the distribution of chemical compartments in a 3D volume, we further developed SRP tomography by collecting a series of SRP images while rotating the sample, and reconstructing the 3D structure through image reconstruction algorithms, as illustrated in Fig. 1c.

### Theory of SRS signal generation by Bessel beams

Assuming collinearly overlapped pump and Stokes beams, the SRS intensity from a very thin slab centred at position **z** with a thickness Δ*z* can be expressed as ref. 30


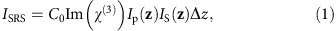


where *C*_0_ is a constant, Im(*χ*^(3)^) is the imaginary part of the third-order nonlinear susceptibility *χ*^(3)^, and *I*_p_(**z**) and *I*_S_(**z**) are the intensities of the pump and Stokes beams at **z**, respectively.

Several methods were reported to generate a Bessel beam^31^,^32^,^33^. Here we used two axicons and an objective lens to convert a Gaussian beam into a Bessel beam (Fig. 1d). In our implementation, a Gaussian beam was first converted to a ring-shaped beam by the axicon pair and then to a Bessel beam via the objective lens. The intensity distribution of the Bessel beam can be modelled as


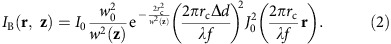


Here (**r**, **z**) are, respectively, the coordinates in the lateral and longitudinal directions, *I*_0_ is the intensity at the centre of the incident Gaussian beam with a half width *w*_0_, 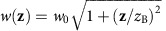, *z*_B_ is the equivalent Rayleigh length of the Bessel beam, *r*_c_ is the radius of the ring-shaped beam, Δ*d* is the width of the ring, *λ* is the wavelength and *f* is the focal length of the objective lens.

By expressing the pump and Stokes beams using the Bessel beam expression in equation (2), we obtain the distribution of SRS intensity


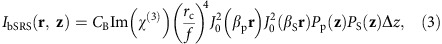


where *C*_B_, *β*_p_ and *β*_S_ are constants, *P*_p_(**z**) and *P*_S_(**z**) are the powers of the pump and Stokes beams, respectively, *J*_0_(·) is the zeroth-order Bessel function.

For a thick sample, the SRP signal intensity is defined as the integration of *I*_bSRS_(**r**, **z**) over the sample thickness *L*


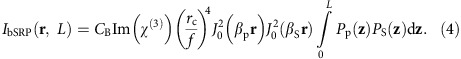


The overall SRP signal is then


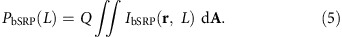


Here **A** is the signal integration area and *Q* is the detection efficiency of the photodetector. The derivations of equations (1, 2, 3, 4, 5) are detailed in Supplementary Notes 1–3.

### Simulation results of the SRP signal

**Figure 2:**
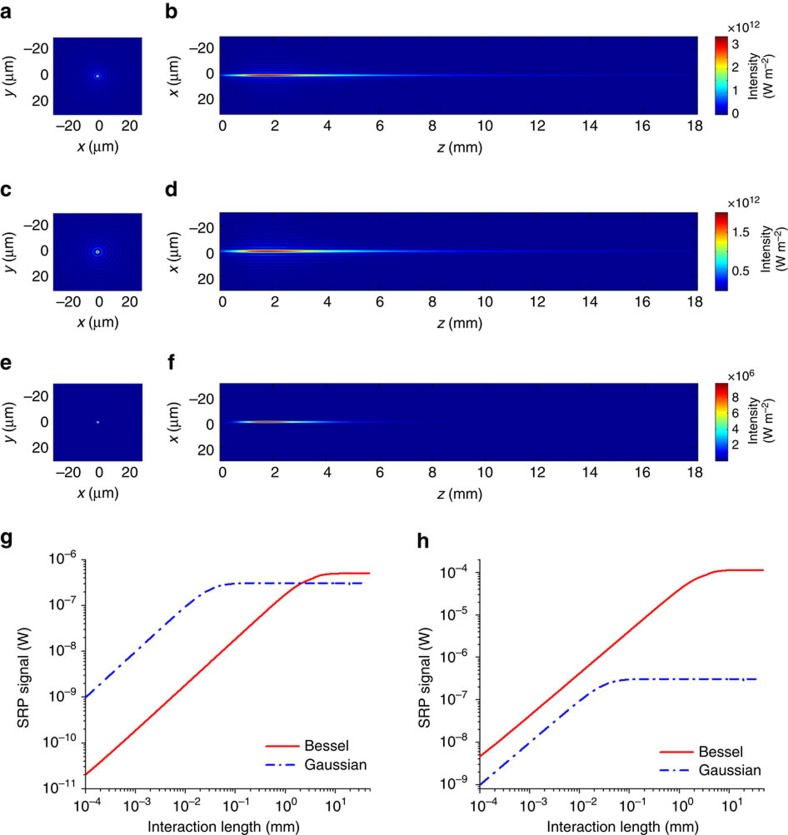
Numerical simulations of the Bessel beam intensity distributions and the stimulated Raman projection signal level. (**a**) The cross-sectional and (**b**) the longitudinal distributions of the pump beam intensity at the wavelength of 800 nm. The colour bar is the same for **a**,**b**. (**c**) The cross-sectional and (**d**) the longitudinal distributions of the Stokes beam intensity at the wavelength of 1,040 nm. The colour bar is the same for **c**,**d**. (**e**) The cross-sectional and (**f**) the longitudinal distributions of the stimulated Raman scattering (SRS) signal intensity generated around 2,885 cm^−1^. The colour bar is the same for **e**,**f**. (**g**) The stimulated Raman projection (SRP) and the SRS signal level as a function of the sample thickness, assuming the same input laser powers for the Bessel and the Gaussian beams. (**h**) The SRP and the SRS signal level as a function of the sample thickness, assuming the same laser power at the Bessel beam central lobe and the total power of Gaussian beam. The red solid curves indicate the SRP signal generated by the Bessel pump and Stokes beams, and the blue dashed curves indicate the SRS signal generated by the Gaussian pump and Stokes beams.

On the basis of equations (2 and 3), we calculated the intensity distributions of the pump, Stokes and SRS fields. A × 10 objective having a numerical aperture (NA) of 0.3 was used in all calculations. As shown in Fig. 2a–d, our approach can generate laterally diffraction-limited Bessel beams for both pump and Stokes fields, which exhibit a high-intensity central lobe with a series of low-intensity rings. Such a beam profile can maintain a long distance in the direction of laser beam propagation. For example, the central lobe of the Stokes beam (at the wavelength of 1,040 nm) has a lateral diameter of 4.02 μm (Supplementary Fig. 1a), and can maintain over 8.89 mm propagation distance with a full width at half maximum (FWHM) of 3.79 mm (Supplementary Fig. 1b). Although the rings of the pump and Stokes beams can partially overlap (Supplementary Fig. 2a), the laser intensity is much lower in the rings than in the central lobe. Thus, the nonlinear SRP signal generated by the partially overlapped rings can be neglected (Supplementary Fig. 2b). It is reasonable to assume that the SRP signal is only arisen from the overlapped central lobes of the Bessel beams (Fig. 2e,f).

Figure 2g,h shows the SRP signal level as a function of the thickness of the sample placed in the centre of the focus, calculated using equation (5). We found a linear dependence of SRP signal on the sample thickness within 2.2 mm. If the sample thickness exceeds 2.2 mm, such linearity failed. This is because 2.2 mm is the longitudinal FWHM of the SRP signal. The power of the Bessel beam is equally distributed on the central lobe and the rings. Therefore, the energy density at the Bessel beam focus is often much lower than that of the Gaussian beam with the same overall input power. Consequently, the SRP signal generated by the Bessel beams can be much lower than the SRS signal generated by the Gaussian beams, especially when the sample is thin (Fig. 2g). For a thick sample, the signal from SRP can be comparable or even larger than the SRS signal generated by the Gaussian beams, due to the long signal integration length. If we assume that the power on the central lobe of the Bessel beam equals the power at the Gaussian focus, the SRP signal generated by the Bessel beams is always larger than the SRS signal from the Gaussian beams (Fig. 2h).

We further calculated the dependence of the SRP signal on the laser power, which showed a positive linear growth, as seen in the Supplementary Fig. 3. We also investigated the influence of the objective NA and magnification on the Rayleigh length of the Bessel beam, the size of the central lobe, the overall SRP signal and the longitudinal FWHM of the SRP signal. As shown in the Supplementary Fig. 4, a larger SRP signal can be achieved by using objectives with lower magnification and higher NA. Furthermore, for a specific NA, the SRP signal decreased with higher magnification. However, for a specific magnification, there was an optimal NA value to generate the maximum SRP signal for each sample thickness. In addition, we found that both the Rayleigh length of the Bessel beam (Supplementary Fig. 5a) and the longitudinal FWHM of the SRP signal (Supplementary Fig. 5b) decreased with a higher magnification (fix the NA value) or NA (fix the magnification value). For the Bessel beam SRP microscopy, the size of the central lobe determines the system resolution, which was independent on the magnification value (Supplementary Fig. 6a) but inversely dependent on the NA value (Supplementary Fig. 6b). Our simulation results can help select an optimal objective for specific SRP imaging applications.

### The SRP microscope

**Figure 3:**
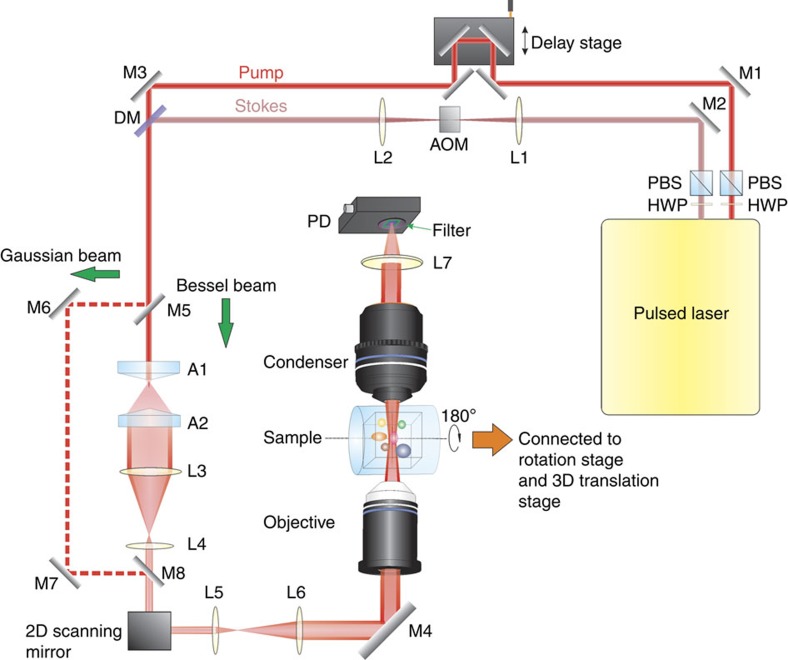
The experimental set-up of the stimulated Raman projection microscopic and tomographic imaging. A tunable pulsed laser provides two synchronized femtosecond pulse trains as pump and Stokes beams. The Stokes beam is modulated by an AOM. The pump beam is first delayed by a translational stage, and is then spatially and temporally combined with the Stokes beam by a DM. The collinearly overlapped beams are sent to a pair of axicons for generating ring-shaped beams. By adjusting the beam size, the ring-shaped beams are first directed to a 2D galvo system for laser scanning, and then guided to an objective for Bessel beams generation. After the sample, the transmitted Bessel beams are collected by a condenser, and then directed to the PD. A pair of shortpass filters are fixed in front of PD to remove the Stokes beam composition. The photocurrent generated in the PD is amplified by a lab-built resonant amplifier, and then sent to a lock-in amplifier for signal extraction. A, axicon; AOM, acousto-optic modulator; DM, dichroic mirror; HWP, half wave-plate; L, lens; M, mirror; PBS, polarizing beam splitter; PD, photodiode.

A schematic of our SRP microscope is depicted in Fig. 3. Two synchronized beams were generated from a pulsed laser (InSight DeepSee, Spectral Physics) at 80 MHz repetition rate. One beam had a fixed wavelength at 1,040 nm and was used as the Stokes beam. The other beam had a tunable wavelength from 680 to 1,300 nm and was used as the pump beam. The Stokes beam was modulated at 2.5 MHz by an acousto-optic modulator (1205-C, Isomet). The pump beam was first delayed by a translational stage, and was then spatially and temporally combined with the Stokes beam by a dichroic mirror. Two axicons (AX2520-B, Thorlabs) were used to convert the overlapped Gaussian beams into ring-shaped beams. After reducing the beam size by a 4-*f* lens system, the ring-shaped beams were directed to a 2D galvo system (GVS012, Thorlabs) for laser scanning. After reflecting by the 2D galvo system and expanding by another 4-*f* lens system, the scanned ring-shaped beams were guided to an objective lens to generate Bessel beams.

The 4-*f* lens system was set up to create a conjugate plane of the galvo system at the entrance of the objective and to expand the ring-shaped beams to match the entrance pupil of the objective lens. After the sample, we used a × 60 water immersion objective (UPlanSApo × 60, NA=1.2, Olympus) to collect the transmitted Bessel beams. Such a high NA objective ensured a high signal collection efficiency to prevent image background from cross-phase modulation. The transmitted beams were first filtered by a pair of shortpass filters (ET980SP-2P, Chroma) to remove the Stokes beam composition and then directed to a large area silicon photodiode (S3994-01, Hamamatsu). The photocurrent generated in the photodiode was first amplified by a lab-built resonant amplifier, and was then sent to a lock-in amplifier (SRS844, Stanford Research Systems, or MFLI, Zurich Instruments) for further amplification and signal extraction.

To facilitate the comparison between the Gaussian beam SRS microscope and the Bessel beam SRP microscope, we built an additional optical arm to allow the laser beams to bypass the axicons, as shown by the dashed lines in Fig. 3. By using flip mirrors, the system can quickly switch between the Bessel SRP and the Gaussian SRS schemes.

### Performance of the SRP microscope

We first verified the simulation results of Bessel beams using a × 10 objective with NA=0.3. For the Stokes beam at the wavelength of 1,040 nm, the diameter of the central lobe and the axial FWHM of the longitudinal propagation were measured to be 4.13 μm and 3.34 mm, respectively (Supplementary Fig. 7). These values closely matched the calculations.

**Figure 4:**
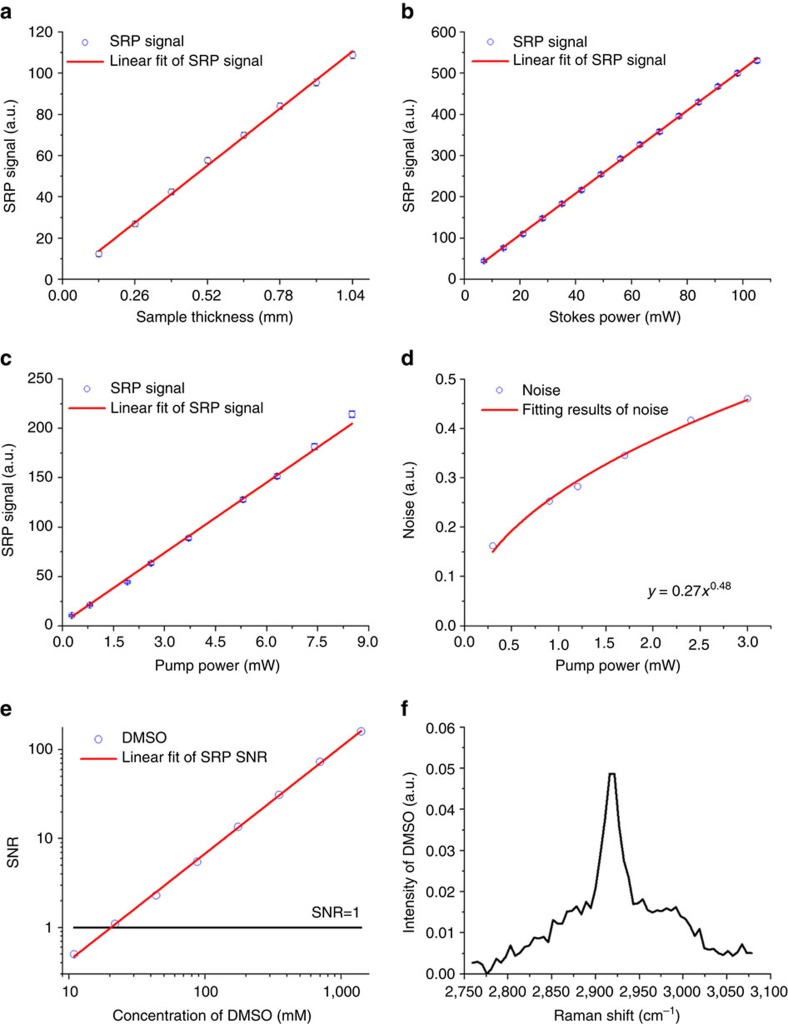
Characterization of the stimulated Raman projection microscope. (**a**) The measured stimulated Raman projection (SRP) signal from PDMS films as a function of the sample thickness. The red line is the linear fitting curve for the measured data points (*R*^2^=0.9977). (**b**,**c**) The SRP signal of PDMS as a function of the Stokes power (**b**) and the pump power (**c**). The red lines are the linear fitting curves for the measured data points (*R*^2^=0.9980 for the Stokes power and *R*^2^=0.9998 for the pump power). (**d**) The SRP noise as a function of the pump power. The red curve is the fitting result of the measured data points (*R*^2^=0.9931). (**e**) The signal-to-noise-ratio (SNR) of the SRP microscopy as a function of sample concentration. The red line is the linear fitting curve of the measured data points (*R*^2^=0.9994). (**f**) The SRP spectrum of DMSO in D_2_O at a concentration of 21.84 mM.

Next, we characterized the performance of the SRP microscope. To investigate the dependence of SRP signal on the sample thickness and the input laser powers, we prepared polydimethylsiloxane (PDMS) films with different thicknesses. Figure 4a shows that the SRP signal increased linearly as the sample thickness increases within millimetre range (*R*^2^=0.9977). Furthermore, the SRP signal was linearly dependent on the powers of both the pump (*R*^2^=0.9998) and the Stokes (*R*^2^=0.9980) beams (Fig. 4b,c). The noise, on the other hand, was proportional to the square root of the pump power (Fig. 4d), indicating a laser shot-noise-limited signal detection.

We then evaluated the sensitivity of the SRP microscope by measuring the CH_3_ symmetric stretching at 2,915 cm^−1^ from dimethyl sulfoxide (DMSO) diluted in deuterium oxide (D_2_O). The sample thickness was 2 mm, smaller than the longitudinal FWHM of the Bessel beams generated by the × 10 objective. The pump and Stokes powers on the central lobe of the Bessel beams were 0.7 and 30 mW, respectively. Figure 4e shows the measured signal-to-noise ratio as a function of DMSO concentration. These results indicated an estimated detection limit of 21.84 mM DMSO from single frequency SRP measurement. From the spectrum measurement, which can further improve the sensitivity of the system, we can clearly resolve the peak from 21.84 mM DMSO (Fig. 4f) and reach a detection limit of 1.8 mM DMSO. We also measured the C=C Raman transition at 1,583 cm^−1^ from retinoic acid, which gave the detection limit as low as 100 μM (Supplementary Fig. 8). This result proved a micro-molar sensitivity of our SRP microscope in the Raman fingerprint region.

### SRP microscopic imaging

**Figure 5:**
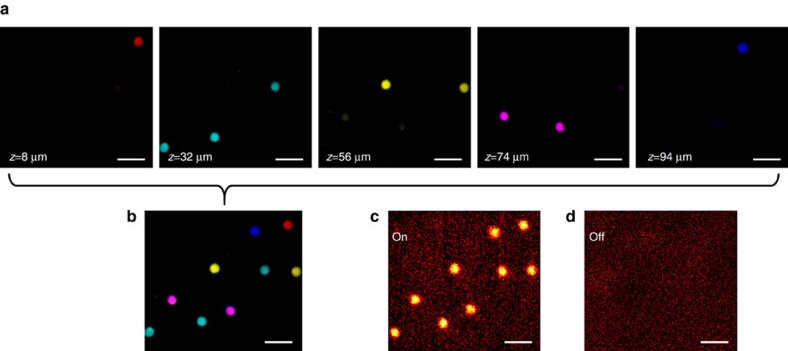
Volumetric imaging of polystyrene beads. (**a**) Sectional images acquired by the Gaussian beam stimulated Raman scattering (SRS) microscope at different depths of the sample volume. (**b**) Superposition of the sectional images in **a**. The false-colours were used to represent beads located at different depths. Stimulated Raman projection (SRP) images of the same beads acquired when the Raman resonance was on (**c**) and off (**d**). The pixel dwell times were 10 μs for the SRS imaging and 50 μs for the SRP imaging. Scale bars, 30 μm.

To prove the capability of our SRP microscope in fast quantification of chemicals in a volume, we imaged polystyrene beads dispersed in a 3D matrix of agarose gel and compared the SRP images with SRS sectioning images obtained from the Gaussian scheme. From the Gaussian beam SRS images, only a portion of the beads can be seen in each sectional plane (Fig. 5a). To image all the beads in the 3D volume, a series of sectional images (50 images, 2 μm per step, as shown in Supplementary Movie 1) were acquired within 20 s. A superposition of the sectional images from selected depths is shown in Fig. 5b. In the SRP image acquired at the same location, all beads in the volume can be resolved by a single 2D lateral scan, finished in 2 s (Fig. 5c,d), an order of magnitude faster than the sectional imaging. Such a speed advantage would be magnified for imaging an even larger volume. We also imaged a mixture of polystyrene beads having 10 and 100 μm diameters (Supplementary Fig. 9). The SRP signal intensity from the 100 μm bead was found to be roughly 10 times as that of the 10 μm beads (both measured at the centre part of the beads). This result further confirmed the validity of our SRP microscope in chemical quantification.

**Figure 6:**
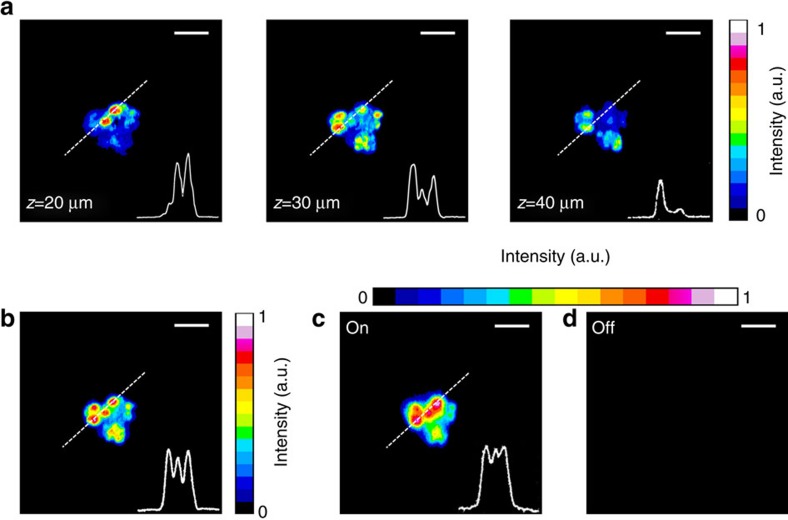
Volumetric imaging of a single adipose cell. (**a**) Sectional images acquired by the Gaussian beam stimulated Raman scattering (SRS) microscope at different depths of the sample. (**b**) Superposition of the 50 sectional images. Stimulated Raman projection (SRP) images of the same cell acquired when the Raman resonance was on (**c**) and off (**d**). The pixel dwell time was 10 μs for both the SRS and SRP imaging. The signal profiles along the dashed lines were display at the bottom-right of the images. Scale bars, 30 μm.

As an example to quantify biomolecules, we imaged lipid droplets (LDs) in differentiated 3T3-L1 cells. We tuned the Raman transition to CH_2_ symmetric vibration at 2,850 cm^−1^ to image the LDs. The cells have near-spherical shapes with diameters up to tens of micrometres. We first acquired 50 sectional images of a cell by the Gaussian beam SRS microscope at 1 μm per depth step. The total image acquisition time was 45 s. Figure 6a shows the sectional images at depths of *z*=20, 30 and 40 μm, displaying very different intracellular morphologies about LDs. Figure 6b gives a superposition of the 50 sectional images, representing the overall lipid content in the cell. The SRP image of the same cell, on the other hand, directly revealed such global information of lipids through a single 2D lateral scan taken only 0.9 s (Fig. 6c,d), giving the morphology and intensity profile similar to those of the superposition image (Fig. 6b). This example further highlighted the advantage of speed in using the SRP microscopy to quantify global information of biomolecules in a volume.

### SRP tomographic imaging

Although the SRP microscopy can quantify chemicals or biomolecules in a volume at a high speed, it loses axial resolution. To solve this limitation, we developed an SRP-based tomographic method to reconstruct the 3D distribution of biomolecules in a volume with optical resolution. In this tomographic method, projection images were collected while rotating the sample at 1° per step for 180 steps. The 180 projection images were then assembled to reconstruct the 3D information within the imaging volume. We first wrote the reconstruction code and then validated the code using simulation. The simulation model contained four objects that generated signals, as shown in Supplementary Fig. 10. The SRP image stack was formed by rotating the sample around the principle axis of the cylindrical volume (along *z* direction) while projecting the objects to a plane along the axis. The location and distribution of the objects were then reconstructed from the SRP image stack with the filtered back-projection (FBP) algorithm^34^. The reconstructed 3D volume and the relevant cross-sectional images from the transverse, coronal and sagittal views are shown in Supplementary Movie 2 and Supplementary Fig. 11, showing good agreement with the original objects (Supplementary Fig. 10).

**Figure 7:**
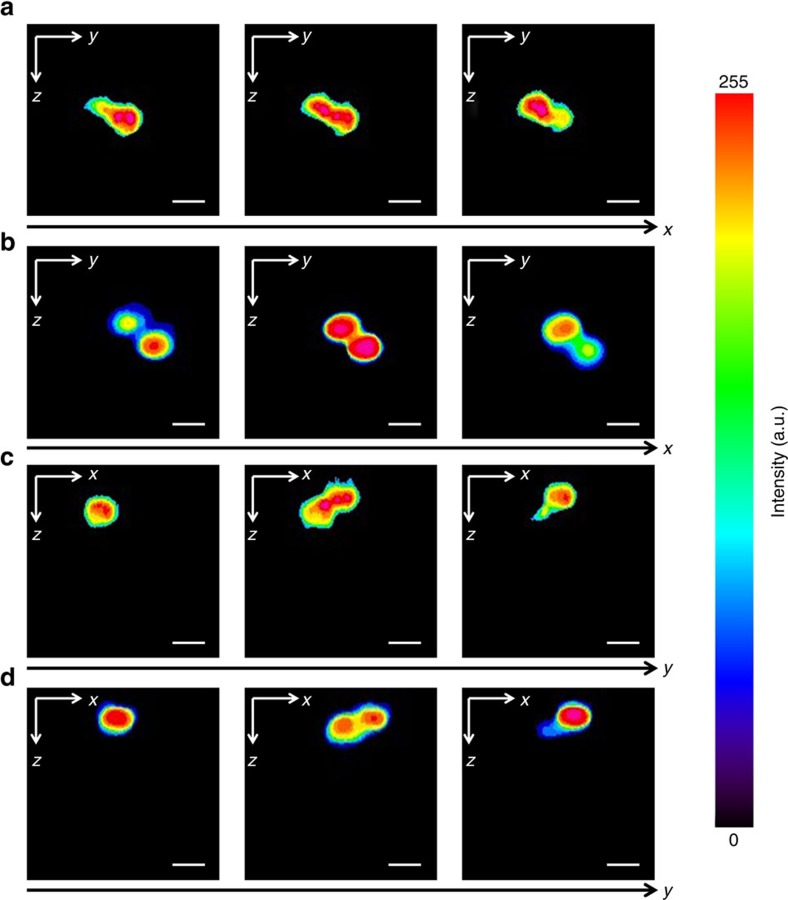
Comparing volumetric imaging results from stimulated Raman projection tomographic and stimulated Raman scattering sectional imaging. (**a**,**b**) Selected image slices of PMMA beads at different positions in the sagittal view (*yz* plane). (**c**,**d**) Selected image slices from the same beads in the coronal view (*xz* plane). (**a**,**c**) Images reconstructed by the stimulated Raman projection (SRP) tomography. (**b**,**d**) Sectional images collected by the Gaussian beam stimulated Raman scattering (SRS) microscope. The imaged volume was ∼60 × 60 × 50 μm^3^ for SRS sectioning imaging and 60 × 60 × 60 μm^3^ for SRP tomographic imaging. The pixel dwell time was 10 μs for the SRS sectional imaging and 24 μs for the SRP tomographic imaging. Scale bars, 10 μm.

Experimentally, we imaged 3D distribution of poly(methyl methacrylate) (PMMA) beads (10 μm) in agarose gel. The laser was tuned to excite Raman transition at 2,950 cm^−1^ from CH_3_ vibration. The sample was loaded into a cylindrical capillary tube (50 μm in diameter) that was fixed onto a rotation stage. The sample was consecutively rotated 180° at 1° per step. The SRP images from the beads were then collected at each step. The reconstructed volume had a dimension around 60 × 60 × 60 μm^3^. In the reconstructed tomographic image, the dimension and location of the beads were well resolved (Supplementary Movie 3). Furthermore, the validity of the tomographic reconstruction was verified by the reconstructed volume from sectional images obtained by the Gaussian beam SRS sectioning imaging on the same beads. The reconstructed 3D volume of the PMMA beads by the sectioning imaging is shown in Supplementary Movie 4. For a direct comparison, we selected the corresponding sections (in coronal and sagittal views) of the beads imaged using both methods, as shown in Fig. 7. Though slightly decreased in spatial resolution, the SRP tomographic results agreed well with the SRS sectioning imaging results in terms of the morphology and localization. The spherical shape of the beads can be well reconstructed with little distortion, which was proved by comparing the bead profiles along three axial directions, as shown in the Supplementary Fig. 12.

The SRP tomography has a greater advantage in imaging speed when a larger volume is measured. As an example, we imaged a 100 μm polystyrene bead in a volume of ∼320 × 320 × 320 μm^3^. The reconstructed 3D volume by the SRP tomography is shown in Supplementary Movie 5. The reconstruction fidelity of the SRP tomography was again confirmed by comparing one of the 3D sectional images with the corresponding one acquired by the Gaussian beam SRS sectioning imaging (Supplementary Fig. 13). Using the SRP tomography, 47 s was needed to collect the SRP image stack of 180 projections. However, using the Gaussian beam SRS sectioning imaging method, it takes 723 sections, equals to 188 s, to complete 3D imaging of such a large volume. In this case, our SRP tomography is four times faster than the sectioning imaging method. For imaging an even larger volume, when more sectional images are needed to reconstruct the volume structure, the advantage of SRP tomography in imaging speed would be further manifested. We note that the imaging speed calculated here is for a single Raman shift. By tuning the wavelength of the pump beam, different Raman shifts can be imaged. The laser tuning can be achieved in tens of milliseconds within the 200 cm^−1^ range or in seconds over a larger range in 4,000 cm^−1^ (ref. 35).

**Figure 8:**
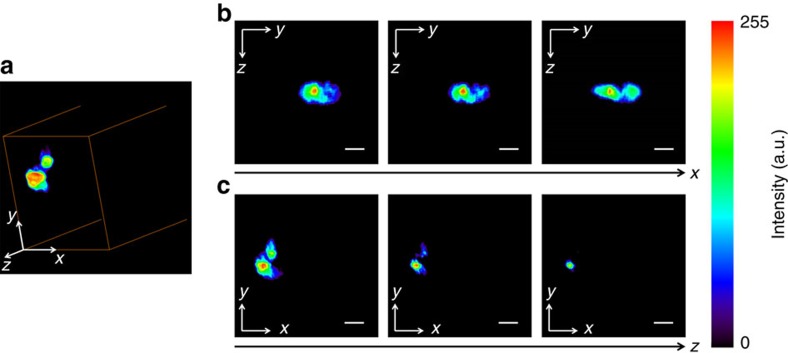
Volumetric imaging of a single adipose cell by stimulated Raman projection tomography. (**a**) Reconstructed 3D structure of a 3T3-L1 cell. (**b**) Selected image slices of the 3T3-L1 cell at different positions in the sagittal view (*yz* plane). (**c**) Images from the same cell in the transverse view (*xy* plane). The imaged volume was ∼150 × 150 × 150 μm^3^. The pixel dwell time was 10 μs. Scale bars, 20 μm.

Next, we performed SRP tomography on single differentiated 3T3-L1 cells to explore the potential of this technique for 3D *in vivo* imaging of biomolecules. The laser was tuned to excite CH_2_ vibration at 2,850 cm^−1^. Figure 8a shows the reconstructed 3D distribution of lipids in the cell, while the complete 3D view is displayed in Supplementary Movie 6. The selected slices in the sagittal and the transverse views displayed different lipid distributions and were shown in Fig. 8b,c, respectively. Such lipid distributions were also revealed in the SRS sectional images in the transverse view (Supplementary Fig. 14), showing good agreement with the tomographic results besides little differences in fine structures, possibly caused by differences in system resolutions, discrepancies in image depths and disagreements in sample orientation. The spatial resolution of the SRP tomographic images was slightly lower than that of the SRS sectional images, caused by the lower effective NA in the SRP measurement (∼0.88 in Bessel SRP and ∼1.07 in Gaussian SRS). These results confirmed the applicability of the SRP tomography for *in vivo* imaging of biomolecules in a 3D volume.

## Discussion

We reported a proof of concept demonstration of the Bessel-beam-based SRP microscopy and tomography for label-free volumetric imaging. Our SRP microscope is capable of quantifying chemical compositions in a living system with a single 2D lateral scan. With the help of the tomographic strategy, we resolved 3D distribution of chemicals in a volume with isotropic spatial resolution. We demonstrated the potential of this new technology by volumetric imaging of polymer particles in a volume and 3D distribution of lipids in single adipose cells.

It is important to discuss pros and cons of our Bessel-beam-based SRP technology in the context of Gaussian-beam-based SRS microscopy. In SRP, the diffraction-free Bessel beam enables volumetric examination of millimetre-scale specimens. Because we use two Bessel beams for the pump and Stokes fields at two different wavelengths, the signal only arises from the central lobe (Fig. 2e,f; Supplementary Fig. 2), which ensures the diffraction-limited spatial resolution. More importantly, because of the energy exchange between the rings and the central lobe during propagation, Bessel beams are able to maintain the focus through a highly scattered medium^33^. Thus, one can potentially apply our SRP technology to examine subjects buried inside a millimetre layer highly scattered medium. These capabilities are beyond the reach by the Gaussian beam SRS microscopy. On the other hand, we note that only a small portion of the total energy resides in the central lobe, our technology is not sensitive to sub-micrometre features, for which SRS microscopy will offer better performance. Meanwhile, we note that there is plenty of room for further improvement of our SRP microscopy and tomography, as discussed below.

First, we can further increase the detection sensitivity of the system. In this work, the input power of the pump beam was limited by the photo-saturation of the detector. This was because the detector collected all the photons distributed on the central lobe and side rings; however, only the central lobe photons contributed for generating SRP signal. A detector with higher saturation power can further improve the sensitivity of the SRP system. Moreover, the input power of the Stokes beam was limited by the current laser system. A more powerful laser with higher output power for the Stokes beam would allow an increase in the generation of SRP signal, in turn a higher detection sensitivity.

Second, we can further increase the spatial resolution of our SRP technology. In this work, we used a dual-axis scanning galvo system with two large area scanning mirrors. The distance between the two mirrors was so large that they cannot be simultaneously conjugated to the entrance pupil of the objective lens. Consequently, to avoid beam clipping during laser scanning, we shrunk the beam smaller than the objective entrance pupil did. Therefore, the effective NA of our system is usually smaller than the actual NA of the objective. For example, using a × 40, 0.8 NA objective, which ideally allowed a 0.38 μm spatial resolution, the SRP microscopy utilized an effective NA of only ∼0.51, which theoretically gave a 0.73 μm spatial resolution (an experimentally measured resolution was ∼0.83 μm, as shown in Supplementary Fig. 15). In the future, an optimized system allowing a galvo system with shorter distance between the mirrors would further increase the effective NA in the SRP scheme, in turn improving the spatial resolution.

Third, we can further improve the imaging quality and speed of SRP tomography. In current experiments, we used a cylindrical glass tube to mount samples. The refractive index mismatch between the glass tube and the sample would deteriorate the quality of the SRP images, in turn reducing the quality and the resolution of the SRP tomographic images. A better sample-mounting scheme, such as using tubes made of fluorinated ethylene propylene that has a refractive index close to that of the sample, can improve the image quality^36^. The imaging speed of SRP tomography can be improved by applying algorithms that are more efficient. The FBP algorithm used in this work required 180 projections for reconstructing a reasonable 3D image^34^. By combining iteration algorithm and the sparse reconstruction strategy^37^, image reconstruction can be performed with much less projection images, which would improve the imaging speed of our SRP tomography.

Fourth, we assumed a constant central lobe intensity for the Bessel beams throughout the sample in the current SRP microscopy and tomography. In fact, the central lobe of the Bessel beam has non-constant intensity distribution along its maximum propagation distance (Supplementary Figs 1b and 7b). By placing the sample at the centre of focus and ensuring the sample thickness smaller than the FWHM of SRP signal, the assumption of a constant value of central lobe intensity is acceptable (Figs 2g,h and 4a). Furthermore, by adding a weighting factor, which is determined by the longitudinal distribution of the central lobe intensity of Bessel beam, to the back-projection intensity during the tomographic reconstruction, the accuracy and the quality of the reconstructed image can be further improved. Alternatively, a logarithmic axicon would generate a Bessel beam with a more constant central lobe intensity for volumetric imaging^38^.

Finally, we note that the reported SRP imaging system is based on a bulky and relatively expensive femtosecond laser, which limits its potential for broad use and clinical translation. Nevertheless, we anticipate that this limitation can be circumvented through innovations in fibre laser, fibre technology and demodulation strategies. Freudiger *et al*.^39^ and Orringer *et al*.^40^ demonstrated high-quality SRS imaging of clinical samples using a fibre laser and a balanced detector. Such approach can be adopted to reduce the cost and size of our technology. We also note that Bessel beam can be produced using fibres^41^, which could simplify the system and enable endoscopic applications. Finally, the lock-in amplifier can be replaced by a low-cost tuned amplifier^35^,^42^. Collectively, these strategies could potentially reduce the cost, size and complexity of the SRP set-up towards broader applications and clinical use.

## Methods

### Selection of the objective lenses

For different purposes, we generated Bessel beams using different objectives lenses, including a × 10 0.3 NA air objective (RMS10X-PF, Thorlabs), a × 40 0.8 NA water immersion objective (LUMPlanFLN × 40, Olympus), a × 60 1.1 NA water immersion objective (LUMFI × 60, Olympus) and a × 25 1.05 NA water immersion objective (XLPL25XWMP, Olympus). The × 10 objective was employed to verify the simulation results, since the Bessel beam generated by this objective had a larger profile for easy measurement and characterization (Supplementary Fig. 1). The × 40 objective was used for the SRP microscopic imaging of polymer particles and biological samples, since it allowed for a tighter focusing and hence generating the stronger SRP signal (Supplementary Fig. 16). The × 60 objective, which allowed even higher signal to be generated (Supplementary Fig. 16), was used in the SRP tomographic imaging of polymer particles and biological samples. The × 25 objective was used for imaging a larger volume (Supplementary Fig. 13). The detailed comparisons of the Bessel beams generated by the four objectives are listed in Supplementary Table 1.

### Sample holder for the SRP tomography

A lab-built sample holder was used to hold and rotate samples^22^. For the SRP microscopic imaging, the sample holder was comprised of a microscope slide holder and a 3D translational stage. The slide holder (MAX3SLH, Thorlabs) was used to hold the sample fixed between two slides or in a cuvette. The 3D translational stage was used to fine tune the sample position at the focus of the Bessel beams. In the SRP tomographic imaging, three more components were added, including a fibre holder, a rotational stage and an additional 3D translational stage. A cylindrical capillary tube, with one end attached to the fibre holder, while the other inserted into a square capillary tube, was used to store samples. The square capillary tube was attached to the slide holder to confine the sample during rotation. The cylindrical capillary tube was rotated while being confined in the square tube. The projection imaging was performed within the boundary of the square capillary tube. The fibre holder (FPH-S, Newport) was attached to the rotational stage installed on a 3D translational stage for position adjustment. The sample rotation was performed by electronically controlling the rotational stage to rotate at 1° per step for 180°. An SRP image was acquired at each rotational step.

### Characterization of the SRP microscope

To measure the dependence of the SRP signal and the noise on sample thickness and laser power, we used PDMS films with different thicknesses. The sample was prepared by sandwiching PDMS between two glass slides. The PDMS film thickness ranged from 130 to 1,040 μm, with a 130 μm increment. The SRP signal from 2,915 cm^−1^ CH_3_ bond vibration was measured (Supplementary Fig. 17a).

The thickness dependence measurements in Fig. 4a were acquired at a 200 μs pixel dwell time. The central lobe powers of the pump and Stokes beams were 0.9 and 6.3 mW, respectively. Signal from the sample was calculated by the average voltage difference between the presence and the absence of the sample.

The laser power dependence measurements in Fig. 4b–d were measured using a 1 mm-thick PDMS, at a 200 μs pixel dwell time. To investigate the dependence of the SRP signal on the Stokes power, the central lobe power of the pump beam was fixed to 0.6 mW and the central lobe power of the Stokes beam was changed from 0.5 to 7.35 mW, with a 0.5 mW increment. For the measurement of signal dependence on the pump power, the central lobe power of the Stokes beam was fixed to 4.2 mW and the central lobe powers of the pump beam were set to 0.02, 0.06, 0.13, 0.18, 0.26, 0.37, 0.44, 0.52 and 0.60 mW. In the noise analysis, the pump powers at the photodiode were set to 0.3, 0.9, 1.2, 1.7, 2.4 and 3 mW.

Different concentrations of DMSO in D_2_O (10.92, 21.84, 43.75, 87.50, 175, 350, 700 and 1,400 mM) were used for the measurement of system sensitivity. The sample, stored in a quartz cuvette, has a thickness of 2 mm. The wavelength of pump beam was set to 798 nm to excite the DMSO Raman shift at 2,915 cm^−1^ (Supplementary Fig. 17b). The pixel dwell time was 1 ms for all the measurements. The central lobe powers of the pump and Stokes beams were 0.7 and 30 mW, respectively. For the SRS spectral measurement at 21.84 mM DMSO, the central lobe powers of the chirped pump and Stokes beams were ∼1.1 and ∼27 mW, respectively. Using the same method, we measured SRP signal from different concentrations of retinoic acid in D_2_O (0.05, 0.1, 0.25, 0.5, 1, 5 and 25 mM). The pump beam wavelength was tuned to 893 nm to excite the Raman shift at ∼1,583 cm^−1^ (Supplementary Fig. 17c). The pump and Stokes powers at the central lobe of the Bessel beams were 0.9 and 23 mW, respectively. The spectra of DMSO and retinoic acid were measured using spectral focusing method^43^.

### SRP microscopic imaging

For the SRP microscopic imaging of polystyrene beads, the wavelength of the pump beam was tuned to 789 nm to excite the Raman shift at ∼3,058 cm^−1^. The beads were suspended in a 1.5 wt% cured agarose gel to prevent particle movement during image acquisition. The sample mixture was sandwiched between two glass slides with ∼130 μm spacing. For the Gaussian beam modality, 50 sectional images were acquired at 2 μm per step in depth. The powers of the pump and Stokes beams at sample were ∼10 and ∼24 mW, respectively. The pixel dwell time was 10 μs and the image size was 200 × 200 pixels. The total acquisition time of an image stack was ∼20 s, excluding the time for data transfer and storage, and the sample stage moving along the depth direction. In the Bessel beam SRP modality, all the beads were resolved in one projection image obtained from a 2D lateral scan. The central lobe powers of the pump and Stokes beams were ∼1 and ∼30 mW, respectively. The pixel dwell time was 50 μs and the image size was 200 × 200 pixels. The total image acquisition time was ∼2 s.

3T3-L1 cells were also embedded in the cured agarose gel and sandwiched between two glass slides for imaging. The wavelength of the pump beam was tuned to 802 nm, exciting the 2,850 cm^−1^ Raman shift from lipids. We first acquired 50 sectional images at 1 μm per step in depth using the Gaussian beam SRS modality. The powers of the pump and Stokes beams at sample were ∼10 and ∼24 mW, respectively. The pixel dwell time was 10 μs and the image size was 300 × 300 pixels. The total acquisition time of the image stack was ∼45 s, excluding the time used for data transfer and storage, and the sample stage movement along the depth direction. The SRP projection image of the whole cell was acquired with the central lobe powers of pump and Stokes beams being 1 and 34 mW, respectively. The pixel dwell time was 10 μs and the image size was 300 × 300 pixels. The total image acquisition time was ∼0.9 s.

### SRP tomographic imaging

For the SRP tomographic imaging of PMMA beads (10 μm), the wavelength of pump beam was tuned to 796 nm. The beads were first mixed with 1.5 wt% agarose gel, and then the sample mixture was cured in a cylindrical capillary tube (inner diameter: 50 μm; outer diameter: 80 μm). One end of the tube was attached to the fibre holder and the other end of the tube was inserted into a square capillary tube (inner width: 100 μm, wall thickness: 50 μm). The square capillary tube was then fixed on the slide holder to prevent sample movement during rotation. By rotating the capillary tube 1° per step, 180 projection images were collected with 24 μs pixel dwell time. Each image was 150 × 150 pixels. The whole imaged volume was ∼60 × 60 × 60 μm^3^. The central lobe powers of the pump and Stokes beams were ∼1.2 and ∼35 mW, respectively. For image comparison, the sectional image stack collected using the Gaussian modality contains 50 images, which were acquired at 1 μm per step. The total volume acquired in imaging was ∼60 × 60 × 50 μm^3^. The pixel dwell time was 10 μs. The powers of the pump and Stokes beams at sample were ∼13 and ∼25 mW, respectively.

Similarly, we imaged a 100 μm PS bead in a volume of ∼320 × 320 × 320 μm^3^ (Supplementary Fig. 13). The total acquisition time for the SRP image stack was 47 s. The powers of the pump and Stokes beams at the central lobe were ∼3 and ∼35 mW, respectively.

For the SRP tomographic imaging of the 3T3-L1 cells, the sample was prepared using a similar way as the beads. A total of 180 projection images were collected at 1° angle increment with 10 μs pixel dwell time. Each image was 300 × 300 pixels. The whole imaged volume was ∼150 × 150 × 150 μm^3^. The central lobe powers of pump and Stokes beams were ∼1 and ∼30 mW, respectively. The total acquisition time for the tomographic image stack was ∼162 s. The sectional image stack collected using the Gaussian modality contains 90 images, which were acquired at 1 μm per step. The total volume acquired in imaging was ∼150 × 150 × 90 μm^3^. The pixel dwell time and the image size were the same as those used in the SRP tomography. The powers of the pump and Stokes beams at sample were ∼13 and ∼25 mW, respectively. The Gaussian beam has an axial resolution of ∼0.65 μm. To image a volume with 150 μm depth, it requires >230 sectional images.

### Image processing and reconstruction

Data acquisition and storage were performed on a lab-written software based on LabVIEW (National Instruments Corporation). The 2D images were processed and analysed using ImageJ. Parallel-beam-based FBP algorithm written in MATLAB (The MathWorks, Inc.) was used to reconstruct the 3D volume from the SRP images^34^. The total reconstruction time for a volume of 150 × 150 × 150 pixels was ∼50 s by using a personal computer (Inter Core i7-4702HQ CPU at 2.2 GHz, 8 GB RAM). The visualization and analysis of the SRP tomographic volume and the volume created from the sectioning images were accomplished using Amide.

The detailed theory for Bessel beam SRP signal calculation, the materials used in this work and other experimental procedures can be found in Supplementary Notes 1–3 and Supplementary Methods.

### Data availability

The data and codes that support the findings of this study are available from the corresponding author on reasonable request.

## Additional information

**How to cite this article:** Chen, X. *et al*. Volumetric chemical imaging by stimulated Raman projection microscopy and tomography. *Nat. Commun.* **8,** 15117 doi: 10.1038/ncomms15117 (2017).

**Publisher's note:** Springer Nature remains neutral with regard to jurisdictional claims in published maps and institutional affiliations.

## Supplementary Material

Supplementary Information Supplementary Figures, Supplementary Table, Supplementary Notes, Supplementary Methods and Supplementary References.

Supplementary Movie 1 Sectional images acquired by the Gaussian beam SRS microscope.

Supplementary Movie 2 Reconstructed 3D volume in the simulated stimulated Raman projection tomography.

Supplementary Movie 3 Reconstructed 3D volume containing PMMA beads by the stimulated Raman projection tomography.

Supplementary Movie 4 Reconstructed 3D volume containing PMMA beads by the stimulated Raman scattering sectioning imaging.

Supplementary Movie 5 Reconstructed 3D volume containing a 100 μm PS bead by the stimulated Raman projection tomography.

Supplementary Movie 6 Reconstructed 3D volume containing a 3T3-L1 cell by the stimulated Raman projection tomography.
