# Supplementary material for: Volumetric chemical imaging by stimulated Raman projection microscopy and tomography
Source: Nat Commun. 2017 Apr 24;8:15117. doi: 10.1038/ncomms15117 (PMC5413981; doi:10.1038/ncomms15117)
Supplement: Supplementary Information — Supplementary Figures, Supplementary Table, Supplementary Notes, Supplementary Methods and Supplementary References. [file ncomms15117-s1.pdf]

Supplementary Note 1 | Stimulated Raman scattering (SRS) generation with two collinear beams

SRS is a third order nonlinear optical process, which probes the third-order nonlinear susceptibility $\chi^{(3)}$ of the sample. It requires a pump beam and a Stokes beam for signal excitation. Typically, when the frequency difference between the two input beams matches the vibrational resonance, energy transfers from photons to molecular vibrations. During this process, the pump field usually experiences a loss (stimulated Raman loss, or SRL) and the Stokes field usually experiences a gain (stimulated Raman gain, or SRG). Laser amplitude modulation and de-modulation scheme is typically applied to detect such SRL or SRG[1-3]. In our work, we measure the SRL of the pump beam. We used a simplified model, assuming the input beams are collinearly overlapped plane waves for the SRS signal generation. In such a case, the evolution of the pump field amplitude can be expressed as[4,5]

$$\frac{dE_{\mathrm{p}}}{d\mathbf{z}} = \frac{6\pi i}{n_{\mathrm{p}}\lambda_{\mathrm{p}}}\chi^{(3)}E_{\mathrm{p}}E_{\mathrm{S}}E_{\mathrm{S}}^{*}, \tag{1}$$

where E_{p} and E_{S} are the electric field amplitudes of the pump and Stokes beams, respectively. $\chi^{(3)}$ is the third-order nonlinear susceptibility of the material, n_{p} is the refractive index of the material at the pump wavelength λ_{p}, i is the imaginary unit, and $\mathbf{z}$ is the axial position in the excitation volume (where the nonlinear SRS process occurs) along the direction of light propagation. The solution of Eq. (1) gives the SRS signal intensity generated from a very thin slab centered at $\mathbf{z}$ with a thickness of Δz

$$I_{\mathrm{SRS}} = C_0 \mathrm{Im}\left(\chi^{(3)}\right) I_{\mathrm{p}}(\mathbf{z}) I_{\mathrm{S}}(\mathbf{z})\Delta z, \tag{2}$$

where C_0 is a constant and has an expression of $C_0 = -2.8 \times 10^4 / (n_{\mathrm{p}} n_{\mathrm{S}} \lambda_{\mathrm{p}})$, n_{S} is the refractive index of the material at the Stokes wavelength λ_{S}, $\mathrm{Im}\left(\chi^{(3)}\right)$ is the imaginary part of $\chi^{(3)}$, $I_{\mathrm{p}}(\mathbf{z})$ and $I_{\mathrm{S}}(\mathbf{z})$ are, respectively, the pump and Stokes beam intensities at $\mathbf{z}$.

Supplementary Note 2 | Theoretical modelling of the Bessel beam generation

An ideal Bessel beam does not diffract or diverge during its propagation[6,7]. In reality, such a non-diffract property of the Bessel beam can maintain a relatively longer distance compared to a Gaussian beam. Due to this unique property, Bessel beams have been successfully used in two-photon fluorescence microscopy[8], selective plane illumination microscopy[9], optical coherence tomography[10], and photoacoustic microscopy[11] for volumetric imaging of a specimen. A number of methods were reported to generate beams with properties reasonably mimicking an ideal Bessel beam, such as using an annular slit[9], an axicon[12], and a spatial light modulator[13]. In this study, we used a pair of axicons and an objective lens to convert a Gaussian beam into a Bessel beam. In our implementation, the axicon pair converted a Gaussian beam to a ring-shaped beam. Such a ring-shaped beam was further converted to a Bessel-Gauss beam after passing the objective (the term ‘Bessel-Gauss beam’ is simplified to ‘Bessel beam’ in the rest of the paper).

This scheme is similar to the case where a Bessel beam is generated using an annual slit. Differently, using the axicon pair, the majority of the laser energy was maintained. We first model the generation of the Bessel beam.

The electric field of Gaussian beam on the first axicon can be expressed as

$$E_{\mathrm{I}}(\mathbf{r},\mathbf{z}) = E_0 \frac{w_0}{w(\mathbf{z})} e^{-\frac{\mathbf{r}^2}{w^2(\mathbf{z})}} e^{i\phi(\mathbf{r},\mathbf{z})}, \tag{3}$$

where $E_{\mathrm{I}}(\mathbf{r},\mathbf{z})$ is the electric field at position $(\mathbf{r},\mathbf{z})$. $\mathbf{r}$ is the radial distance from the central axis of the beam and $\mathbf{z}$ is the axial distance along the beam propagation direction. E_0 is the electric field amplitude,w_0 is the beam waist of the incident Gaussian beam, and $w(\mathbf{z})$ is the beam waist at position $\mathbf{z}$. $\phi(\mathbf{r},\mathbf{z})$ is the phase of the incident Gaussian beam.

The electric field of the ring-shaped beam after the second axicon (before the objective) has an expression

$$E_{\mathrm{R}}(\mathbf{r},\mathbf{z}) = E_0 \frac{w_0}{w(\mathbf{z})} e^{-\frac{\mathbf{r}^2}{w^2(\mathbf{z})}} e^{i\phi(\mathbf{r},\mathbf{z})} \mathrm{rect}(\mathbf{r}-r_{\mathrm{c}}). \tag{4}$$

Here, we introduce a rectangle function to model the ring-shaped beam

$$\mathrm{rect}(\mathbf{r}-r_{\mathrm{c}}) = \begin{cases} 1, \ |\mathbf{r}-r_{\mathrm{c}}| \leq \frac{\Delta d}{2} \\ 0, \ |\mathbf{r}-r_{\mathrm{c}}| > \frac{\Delta d}{2} \end{cases}, \tag{5}$$

where r_{c} and Δd are the radius and the width of the ring, respectively.

The Bessel beam generated after the objective lens can be calculated as the Fourier-Bessel transform of the electric field of the ring-shaped beam[7]. By assuming that the electric field of the ring is uniformly distributed in the radial direction throughout the ring width Δd, and utilizing the Fresnel diffraction formula, the electric field of the generated Bessel beam can be modeled as

$$E_{\mathrm{B}}(\mathbf{r},\mathbf{z}) = E_0 \frac{w_0}{w(\mathbf{z})} e^{-\frac{r_{\mathrm{c}}^2}{w^2(\mathbf{z})}} \left(-i\frac{2\pi}{\lambda}\frac{r_{\mathrm{c}}}{f}\Delta d\right) J_0\left(\frac{2\pi}{\lambda}\frac{r_{\mathrm{c}}}{f}\mathbf{r}\right) e^{i\phi(\mathbf{r},\mathbf{z})}. \tag{6}$$

Here, $E_{\mathrm{B}}(\mathbf{r},\mathbf{z})$ is the electric field of the generated Bessel beam, J_0 is the 0th order Bessel function, $w(\mathbf{z})=w_0\sqrt{1+(\mathbf{z}/z_{\mathrm{B}})^2}$, z_{B} is the equivalent Rayleigh range of the generated Bessel beam which can be determined by its maximum propagation distance (MPD), and f is the focal length of the objective and has the expression

$$f = \frac{F}{M}\frac{NA}{\tan(\mathrm{asin}(NA/n))}. \tag{7}$$

Here, F and M are the focal length of the tube lens and the magnification of the objective lens, respectively. NA is the numerical aperture of the objective lens. n is the refractive index of the immersion material for the objective. To ensure the ring-shaped beam to pass the objective during laser scanning, the size of the ring was smaller than the entrance pupil of the objective was. Thus, the effective NA in SRP is smaller than the NA of the objective, and can be determined using the equation of

$$eNA = n\sin(\mathrm{atan}(\frac{r_c}{f})). \tag{8}$$

The intensity distribution of the generated Bessel beam can be calculated from Eq. (6). Considering $I = n|E|^2/753$, we have

$$I_B(\mathbf{r}, \mathbf{z}) = I_0 \frac{w_0^2}{w^2(\mathbf{z})} e^{-\frac{2r_c^2}{w^2(\mathbf{z})}} \left(\frac{2\pi r_c \Delta d}{\lambda f}\right)^2 J_0^2\left(\frac{2\pi r_c}{\lambda f}\mathbf{r}\right), \tag{9}$$

where I_0 is the intensity at the center of the incident Gaussian beam, and λ is the wavelength of the Bessel beam.

The most important optical property of the Bessel beam is the extended depth of focus (DOF). Here, the DOF is also called MPD for the Bessel beam. Within the DOF, the diameter of the Bessel beam central lobe remains unchanged. Referring to literatures[14,15], the DOF of the Bessel beam is defined as the distance between the $1/e^2$ intensity positions along the beam propagation direction on-axis. The DOF of Bessel beam can be expressed as[7]

$$MPD = \frac{\pi D d_B}{4\lambda}, \tag{10}$$

where D is the effective aperture of the objective. In our case, D equals the diameter of the ring, and d_B is the diameter of the central lobe of the Bessel beam which can be determined by the first positive zero point of the 0th order Bessel function $J_0(2\pi r_c\mathbf{r}/\lambda f)$,

$$d_B = 2.4048 \frac{\lambda f}{\pi r_c}. \tag{11}$$

Rearranging equations and considering the M^2 factor of Bessel beam, Eqs. (10) and (11) can be rewritten as

$$MPD = 1.2024 \frac{1}{M^2} \frac{F}{M} \frac{NA}{\tan(\mathrm{asin}(NA/n))}, \tag{12}$$

$$d_B = 0.7655 \frac{\lambda}{r_c} \frac{F}{M} \frac{NA}{\tan(\mathrm{asin}(NA/n))}. \tag{13}$$

Here, M^2 is the M^2 factor of the generated Bessel beam calculated referring to literatures[14,16].

Considering the relationship between the average power and the intensity distribution, $P = f_{rep}\tau \int I(\mathbf{r}, \mathbf{z})\mathrm{d}\mathbf{A}$, we have

$$I_B(\mathbf{r}, \mathbf{z}) = \frac{1}{f_{rep}\tau} \frac{2\pi}{\lambda^2} \left(\frac{r_c}{f}\right)^2 \frac{1}{\int_0^\infty x J_0^2(x)\,\mathrm{d}x} P(\mathbf{z}) J_0^2\left(\frac{2\pi r_c}{\lambda f}\mathbf{r}\right), \tag{14}$$

where f_{rep} and τ are the repetition rate and the pulse width of the laser, respectively. $P(\mathbf{z})$ is power distribution of Bessel beam along the light propagation direction and has an expression of

$$P(\mathbf{z}) = I_0 \frac{w_0^2}{w^2(\mathbf{z})} e^{-\frac{2r_c^2}{w^2(\mathbf{z})}} \left(\frac{2\pi r_c \Delta d}{\lambda f}\right)^2 f_{rep}\tau \frac{\lambda}{2\pi} \left(\frac{f}{r_c}\right)^2 \int_0^\infty x J_0^2(x)\,\mathrm{d}x. \tag{15}$$

Supplementary Note 3 | Theoretical modelling of the stimulated Raman projection (SRP) generation with two Bessel beams

Expressing the pump and Stokes beams using Bessel expression and substituting them into Eq. (2), we can obtain the intensity distribution of the SRS signal from a very thin slab centered at **z** with a thickness of Δz:

$$I_{\mathrm{bSRS}}(\mathbf{r},\mathbf{z}) = C_{\mathrm{B}}\mathrm{Im}\left(\chi^{(3)}\right)\left(\frac{r_{\mathrm{c}}}{f}\right)^4 J_0^2\left(\beta_{\mathrm{p}}\mathbf{r}\right) J_0^2\left(\beta_{\mathrm{S}}\mathbf{r}\right) P_{\mathrm{p}}(\mathbf{z}) P_{\mathrm{S}}(\mathbf{z})\Delta \mathrm{z}. \tag{16}$$

Here, $C_{\mathrm{B}} = C_0 C_1 C_2$ is a constant, where $C_1 = 1/\left(f_{\mathrm{rep}}^2 \tau_{\mathrm{p}} \tau_{\mathrm{S}} A^2\right)$ and $C_2 = \left(2\pi/\lambda_{\mathrm{p}}\lambda_{\mathrm{S}}\right)^2$ with A being the sectional area of the Bessel beam at focus. $\beta_{\mathrm{p}} = 2\pi r_{\mathrm{c}}/\lambda_{\mathrm{p}} f$ and $\beta_{\mathrm{S}} = 2\pi r_{\mathrm{c}}/\lambda_{\mathrm{S}} f$ are the wavelength related wave vectors.

For a thick sample, the SRP signal intensity is defined as the integration of $I_{\mathrm{bSRS}}(\mathbf{r},\mathbf{z})$ over the sample thickness L. Thus, the final expression of the defined SRP signal intensity is

$$I_{\mathrm{bSRP}}(\mathbf{r},L) = C_{\mathrm{B}}\mathrm{Im}\left(\chi^{(3)}\right)\left(\frac{r_{\mathrm{c}}}{f}\right)^4 J_0^2\left(\beta_{\mathrm{p}}\mathbf{r}\right) J_0^2\left(\beta_{\mathrm{S}}\mathbf{r}\right) \int_0^L P_{\mathrm{p}}(\mathbf{z}) P_{\mathrm{S}}(\mathbf{z}) d\mathbf{z}. \tag{17}$$

Experimentally we integrate the signal intensity and detect the total power. Therefore, the overall SRP signal is

$$P_{\mathrm{bSRP}}(L) = Q \iint I_{\mathrm{bSRP}}(\mathbf{r},L)\mathrm{d}\mathbf{A}. \tag{18}$$

Here **A** is signal integration area, and Q is the detection efficiency of the photodetector.

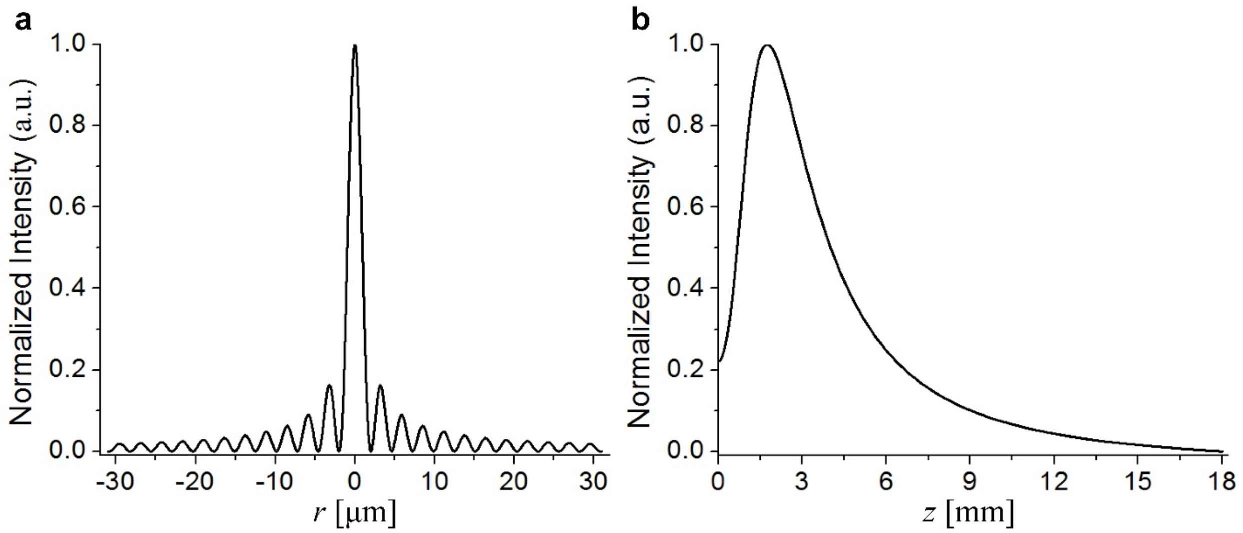

Supplementary Figure 1 | Characterizations of the calculated Bessel beam.

(**a**) The cross-sectional profile of the calculated Bessel beam at a wavelength of 1040 nm. The diameter of the central lobe is ~4.02 µm. (**b**) The longitudinal profile of the maximum propagation distance of the calculated Bessel beam at 1040 nm. The maximum propagation distance is 8.89 mm with a FWHM of 3.46 mm. Here, a 10X 0.3 NA objective was used for simulation.

For detail calculations, we first determined the maximum propagation distance and the central lobe diameter by using Supplementary Equations 12 and 13, and then calculated the longitudinal profile and the cross-sectional profile by using Supplementary Equation 9. The ring radius and width were given as 3.4 mm and 340 µm, and the radius of the incident Gaussian beam was 2.5 mm.

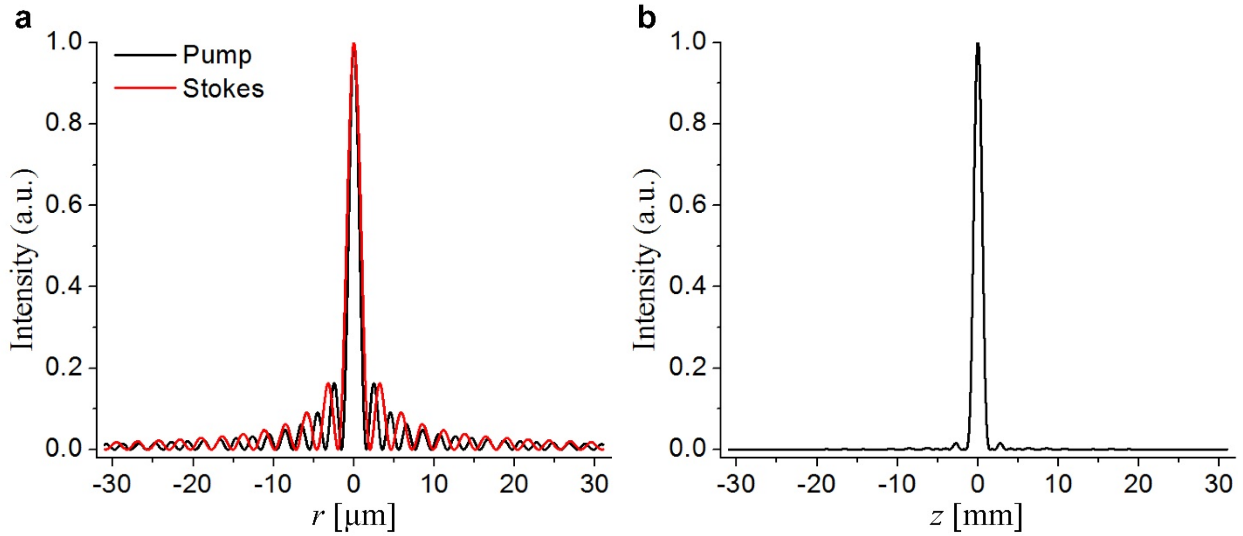

Supplementary Figure 2 | Influence of the Bessel beam ring-overlap on the SRP signal.
(**a**) The cross-sectional profiles of the calculated pump (at the wavelength of 800 nm) and Stokes (at the wavelength of 1040 nm) Bessel beams. (**b**) The cross-sectional profile of the calculated SRP signal generated with the two Bessel beams. These profiles showed that the rings from the pump and Stokes Bessel beams partially overlapped. However, the SRP signal contribution from the rings is less than 2%. Here, a 10X 0.3 NA objective was used for simulation. SRP: stimulated Raman projection.

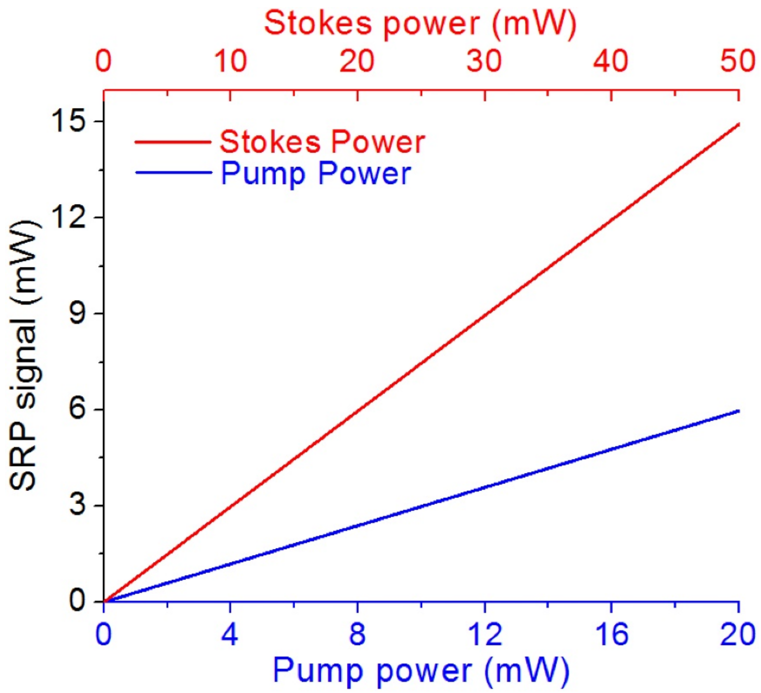

Supplementary Figure 3 | Calculated SRP signal as a function of laser power.
The calculated SRP signal is linearly dependent on the pump (blue solid line) and the Stokes powers (red solid line). Here, a 10X 0.3 NA objective was used for simulation. SRP: stimulated Raman projection.

These results were calculated by using Supplementary Equation 18 with the parameters as: the repetition rate f_{rep} and the pulse width τ for both the pump ($\lambda_p = 800\ nm$) and Stokes beams ($\lambda_p = 1040\ nm$) were 80 MHz and 120 fs; the ring radius r_c was given as 3.4 mm; the thickness of the sample was 2 mm with the imaginary part of the third-order nonlinear susceptibility $\mathrm{Im}\left(\chi^{(3)}\right)$ of 10^{-22}; the detection efficiency Q of the detector was set as 1.

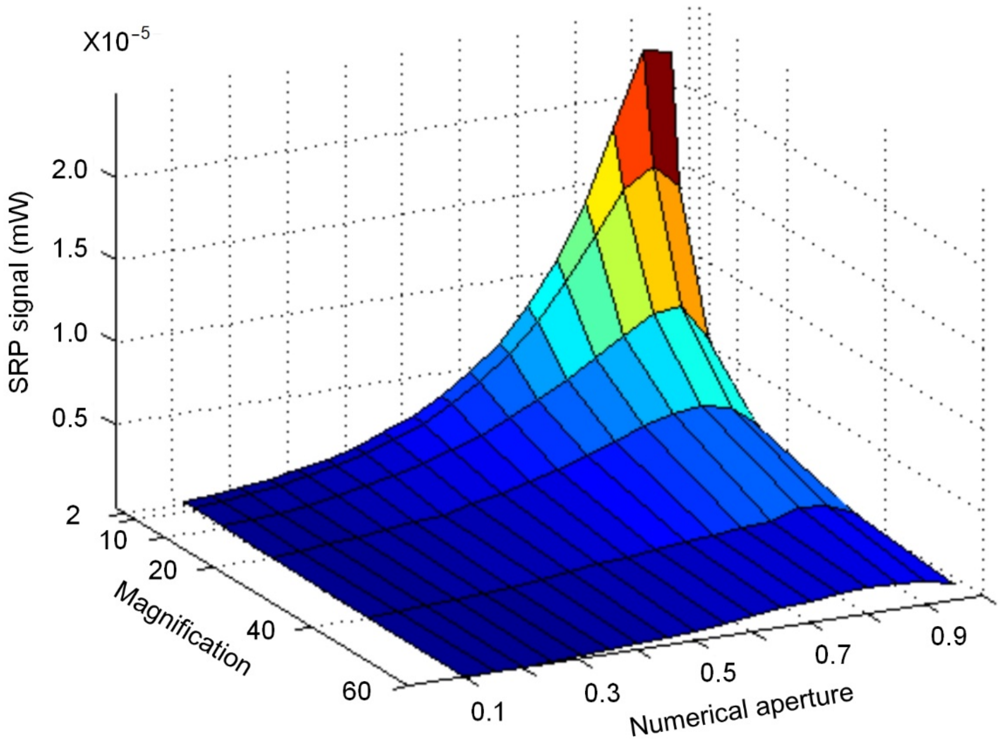

Supplementary Figure 4 | Influence of objective parameters on the calculated SRP signal.

The calculated SRP signal as a function of magnification and NA of the objective. SRP: stimulated Raman projection.

These results were calculated by using Supplementary Equation 18 with the same parameters as those used in Supplementary Figure 3. The difference is that the laser powers of two Bessel beams were changed in the calculation of Supplementary Figure 3, whereas the magnification and NA of the objective were changed here.

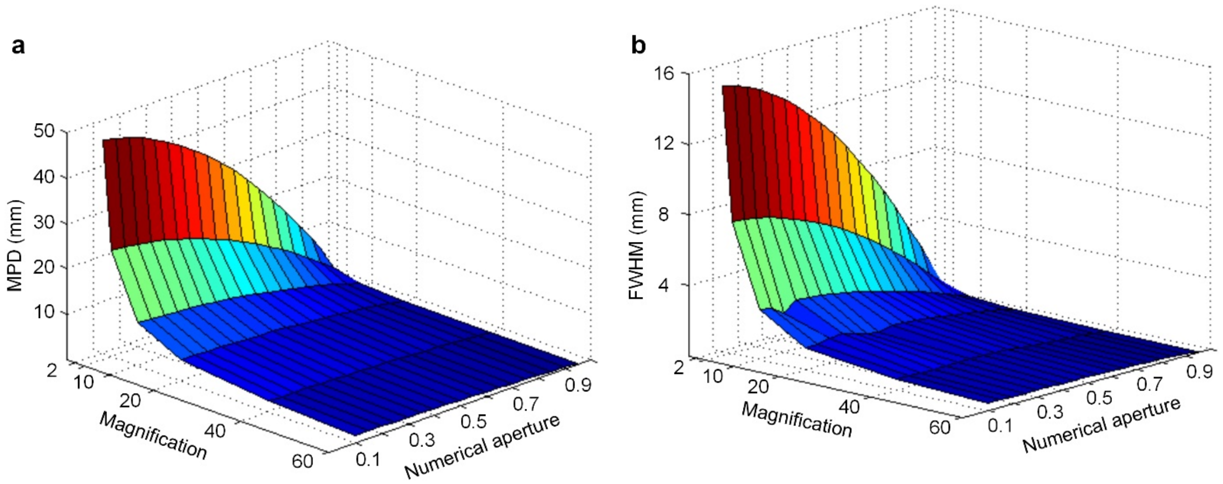

Supplementary Figure 5 | Influence of objective parameters on the calculated depth of focus.

(**a**) The maximum propagation distance of the calculated Bessel beam as a function of NA and magnification of the objective. (**b**) FWHM of the SRP signal as a function of NA and magnification of the objective. The air objective was assumed in all of the calculations. SRP: Stimulated Raman projection.

The results shown in panel (a) were calculated by using Supplementary Equation 12. The related parameters were set as follows:

the M^2 factor was set as 1.2; the repetition rate f_{rep} and the pulse width τ for both the pump ($\lambda_p = 800\,nm$) and Stokes beams ($\lambda_p = 1040\,nm$) were 80 MHz and 120 fs; the ring radius r_c was given as 3.4 mm; the thickness of the sample was 200 μm with the imaginary part of the third-order nonlinear susceptibility $\mathrm{Im}\left(\chi^{(3)}\right)$ of 10^{-22}; the powers of two Bessel beams were set to be unity at position of the peak focus.

The results shown in panel (b) were calculated by using Supplementary Equation 16 with the same parameters as those used in panel (a). The difference is that the thickness of the sample was set to be infinity. The full-width at half-maximum (FWHM) of the SRP signal was determined as:

first, we assumed the radial position to be zero ($\mathbf{r} = 0$) and calculated the generated stimulated Raman signal intensity profile along **z** direction; second, we measured the FWHM of the profile.

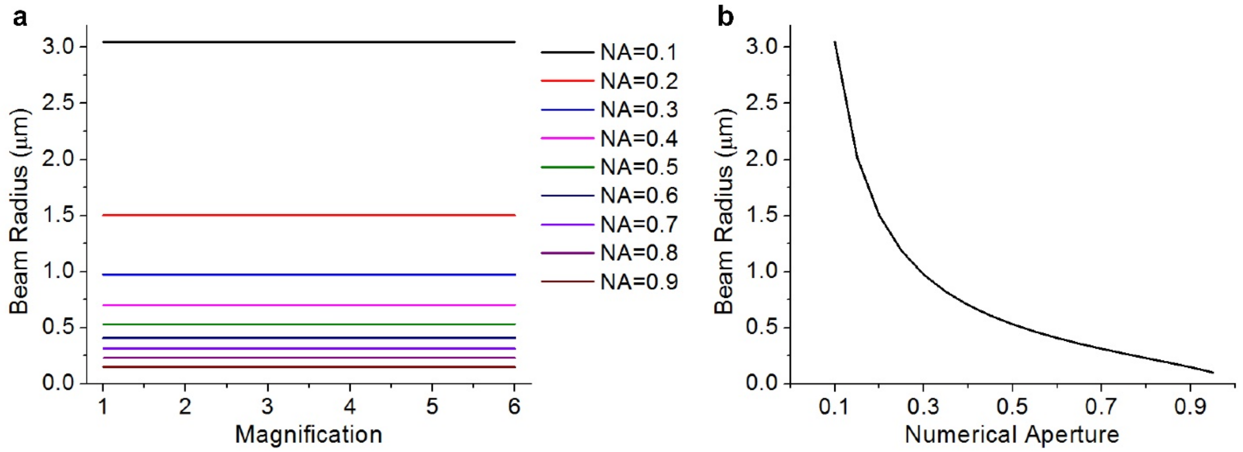

Supplementary Figure 6 | Influence of objective parameters on the size of the Bessel beam central lobe.

(**a**) The calculated central lobe size of the Bessel beam (at the wavelength of 800 nm) as a function of magnification, showing that the central lobe size is independent on objective magnification for a fixed NA. (**b**) The calculated central lobe size of the Bessel beam (at the wavelength of 800 nm) as a function of numerical aperture (NA) of the objective. The central lobe size decreases as the objective NA increases.

These results were calculated by using Supplementary Equation 13. Here, the ring radius r_c was equal to the back aperture of the objective lens.

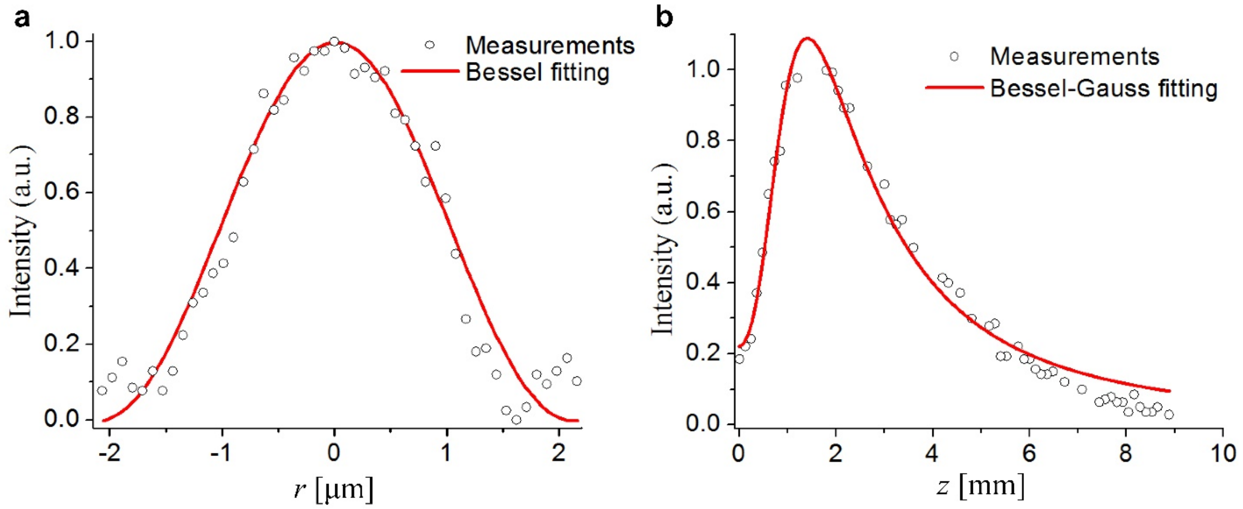

Supplementary Figure 7 | Characterizations of the experimentally generated Bessel beam. (**a**) The beam profile of the Bessel beam central lobe measured perpendicularly across the beam. The measured diameter of the central lobe is about 4.13 μm. (**b**) The longitudinal profile of the Bessel beam central lobe measured along the beam propagation direction. The measured FWHM is around 3 mm. Here the laser beam was at a wavelength of 1040 nm. The objective lens used here was 10X, 0.3 NA. These results were measured by the method of scanning a charge-coupled device (CCD) pixel[17].

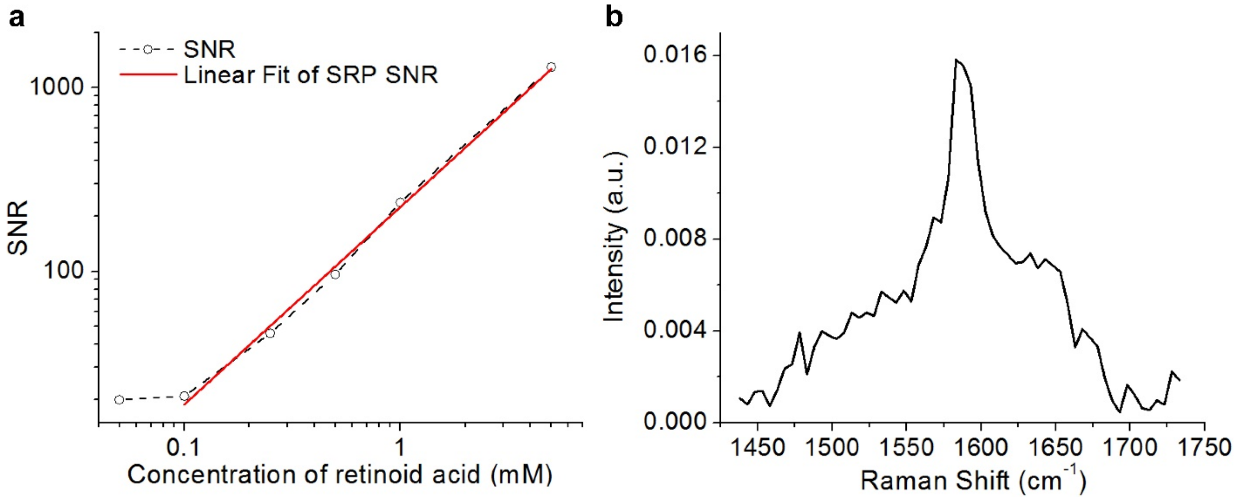

Supplementary Figure 8 | Evaluation of the system sensitivity using retinoic acid.
(**a**) The SNR of the SRP microscopy as a function of the retinoic acid concentration in DMSO, showing a 100 μM detection sensitivity. (**b**) The SRP spectrum of retinoic acid at a concentration of 100 μM. SRP: stimulated Raman projection.

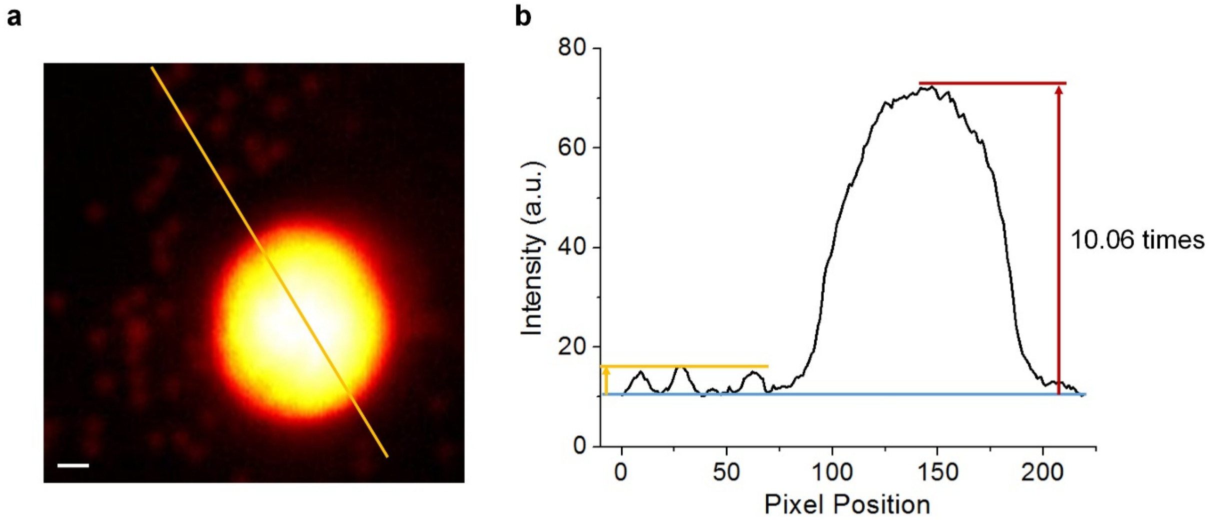

Supplementary Figure 9 | SRP microscopic imaging of a mixture of polystyrene beads.
(**a**) The SRP image of a mixture of polystyrene beads having 10 µm and 100 µm diameters. (**b**) The intensity profile along the line labeled in **a**. The SRP intensity of the 100 µm bead is ~10 times that of the 10 µm beads. The pixel dwell time: 120 µs; pixel number: 200×200. SRP: stimulated Raman projection. Scale bar: 20 µm.

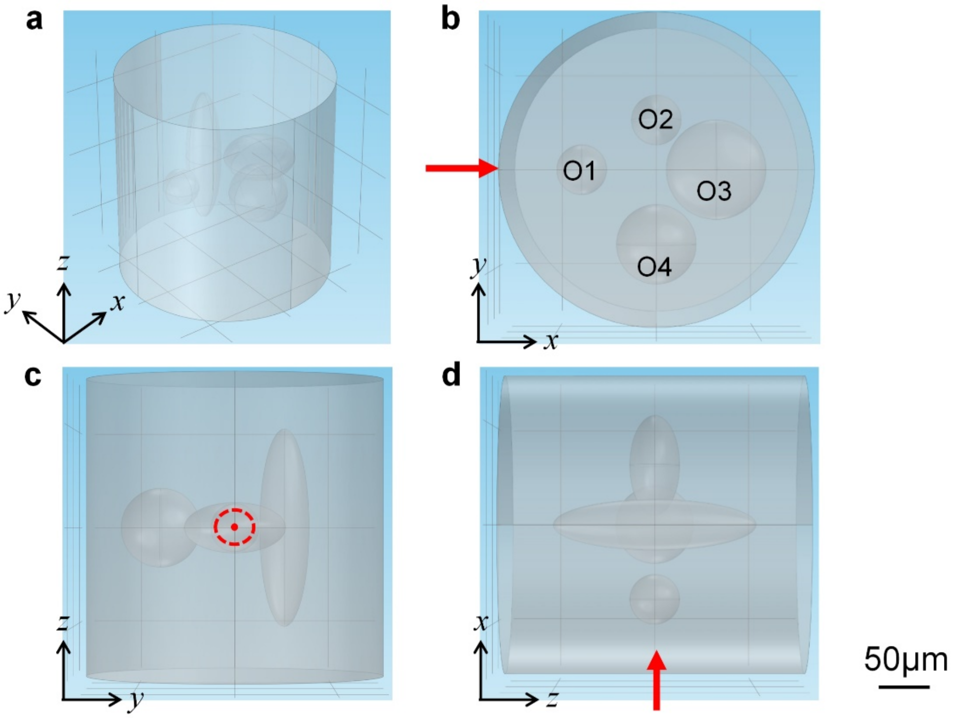

Supplementary Figure 10 | The physical model used in the simulation of the SRP tomography.

(**a**) 3D view of the physical model. (**b-d**) The transverse (**b**), coronal (**c**), and sagittal (**d**) views of the physical model. The red arrows and the dotted circle show the position and direction of the incident laser beams. The physical model was created by using the software package of COMSOL Multiphysics (COMSOL Inc.). SRP: stimulated Raman projection.

The simulated projection images were obtained as follows:

First, we calculated the overall SRP signal along an excitation point using Supplementary Equation 18 (Equation 5 in main text).

Second, we moved the physical model in both x and y directions to form a two-dimensional SRP image (101×101 pixels).

Last, we rotated the physical model along z direction with a step of 1° to obtain an image stack of 180 projections.

The imaginary part of the third-order nonlinear susceptibility $\mathrm{Im}(\chi^{(3)})$ for four objects (O1, O2, O3, and O4) were set as 2×10^{-23}, 1.5×10^{-23}, 1.5×10^{-23}, and 1×10^{-23} respectively.

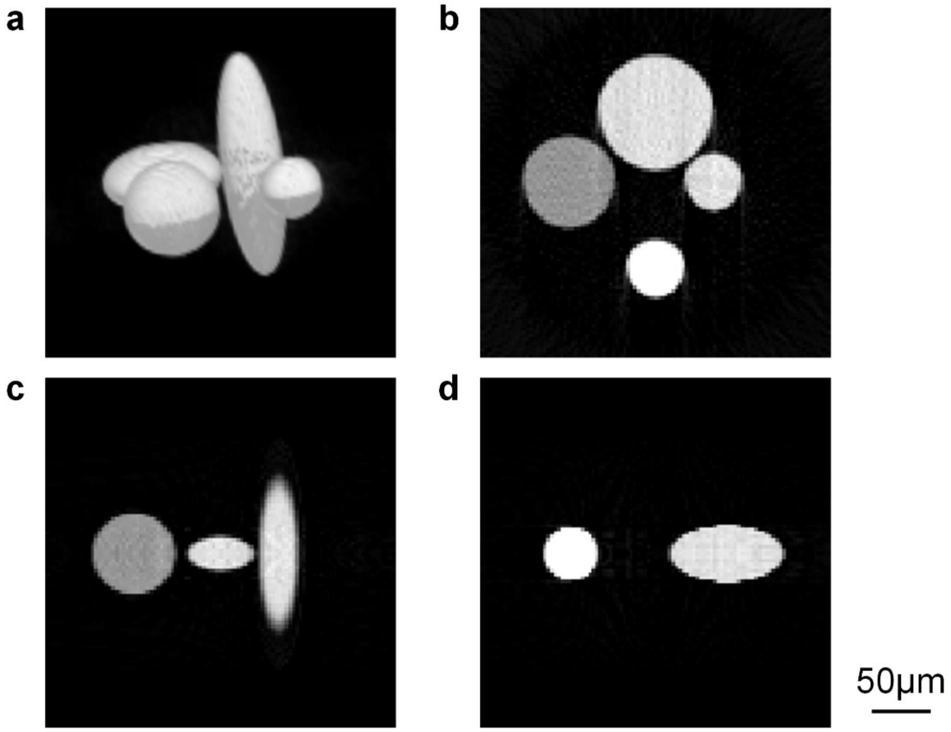

Supplementary Figure 11 | Reconstructed results for the simulated objects by the SRP tomography.

(**a**) Reconstructed 3-D volume. (**b**-**d**) The cross-sectional images from the transverse (**b**), coronal (**c**), and sagittal (**d**) views. SRP: stimulated Raman projection

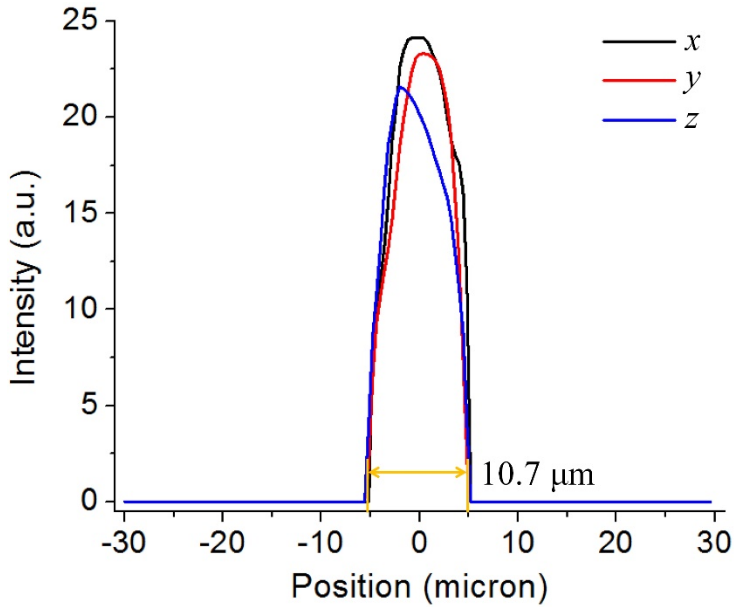

Supplementary Figure 12 | Axial profiles of the reconstructed bead by the SRP tomography. The selected profiles from the central plane of one of the 10 μm beads along three axial directions. The widths of the profiles are 10.58, 10.77, 10.74 μm in x, y, and z axes, respectively. SRP: stimulated Raman projection

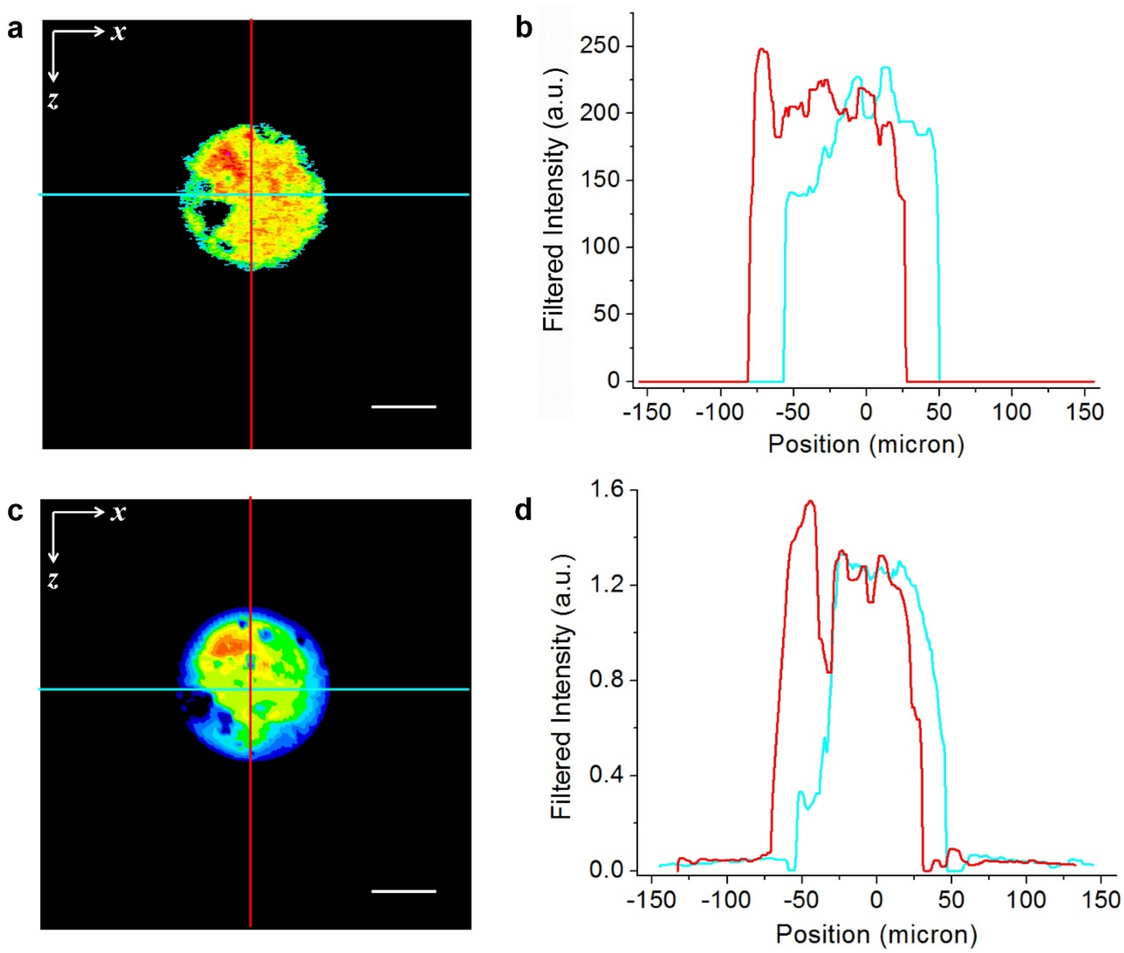

Supplementary Figure 13 | Comparisons of the SRP tomography and the SRS sectioning imaging on a large volume.

(**a**) The central slice selected from the reconstructed volume of a 100 μm polystyrene bead by the SRP tomography. (**b**) The intensity profiles along the lines in the panel (a). (**c**) The sectional image from the same bead acquired by the Gaussian beam SRS microscope. (**d**) The intensity profiles along the lines in the panel (c). The pixel dwell time was 2 μs for both the SRP and SRS images. SRP: stimulated Raman projection; SRS: stimulated Raman scattering. Scale bar: 50 μm.

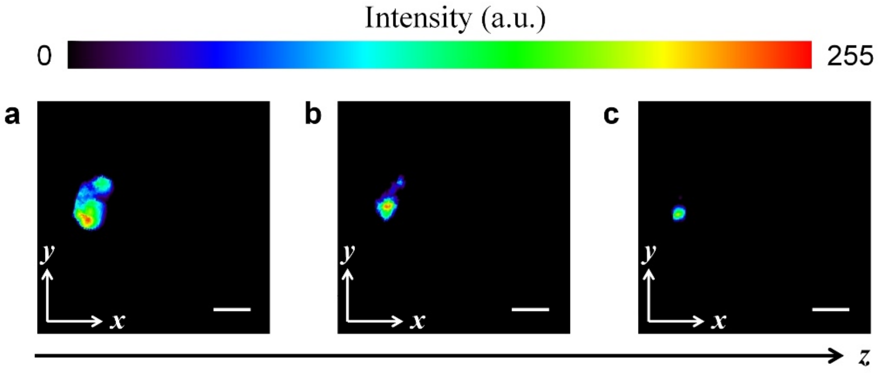

Supplementary Figure 14 | SRS sectional images of the 3T3-L1 cell.

(**a-c**) Selected sectional images of the 3T3-L1 cell acquired by the Gaussian beam SRS microscope in the sagittal view. This is the same cell as shown in Figure 8. SRS: stimulated Raman scattering. Scale bar: 24 μm.

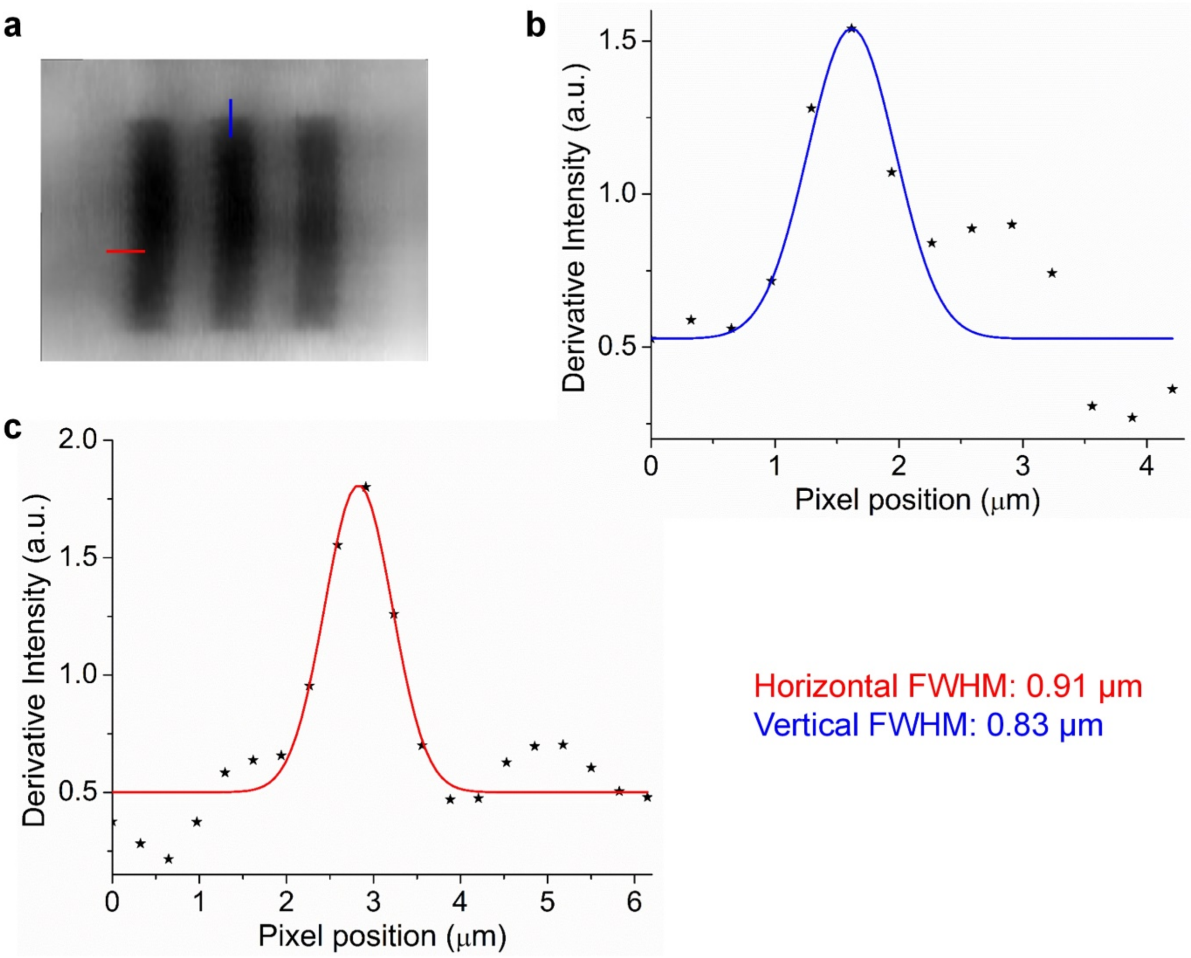

Supplementary Figure 15 | Spatial resolution of the SRP microscope.

(**a**) SRP transmission image of the Element 4 in Group 6 on a negative USAF 1951 resolution target. The 1st order derivative of intensity profiles of the vertical (blue) and horizontal (red) lines were plotted at the panel (**b**) and the panel (**c**). Dots are experimentally measured data and lines are Gaussian-fitting results. The FWHM was determined to be 0.91 μm horizontally and 0.83 μm vertically. The measurements were obtained using a 40X 0.8 NA objective (effective NA was ~0.51). SRP: stimulated Raman projection.

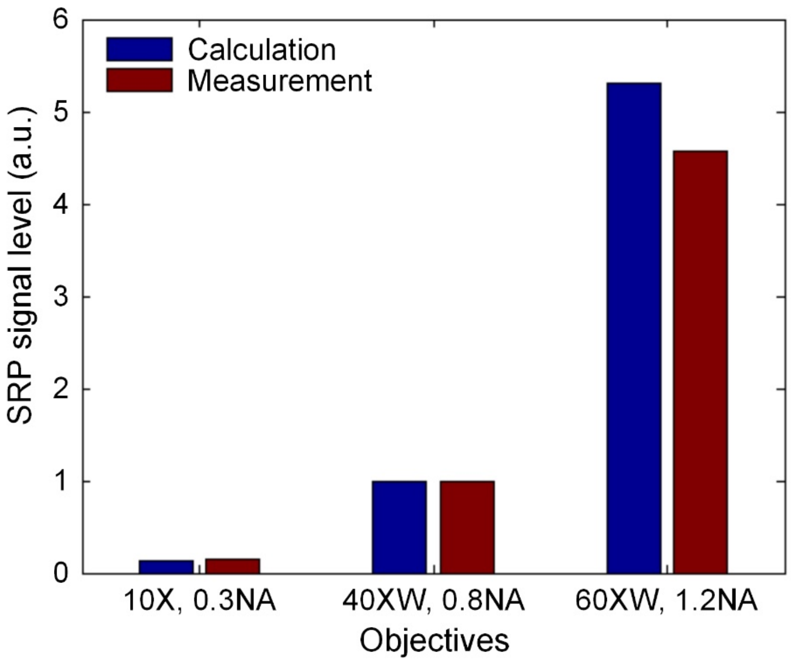

Supplementary Figure 16 | The calculated and measured SRP signal generated by different objectives.

SRP signals generated by different objectives, including (10X, 0.3NA), (40XW, 0.8NA), and (60XW, 1.2NA). The blue bars show the theoretically calculated results, and the red bars are the measured results by our SRP microscope. The SRP signal was normalized by the values obtained from the 40X water immersion objective. The sample used here was a 200 μm olive oil film. SRP: stimulated Raman projection.

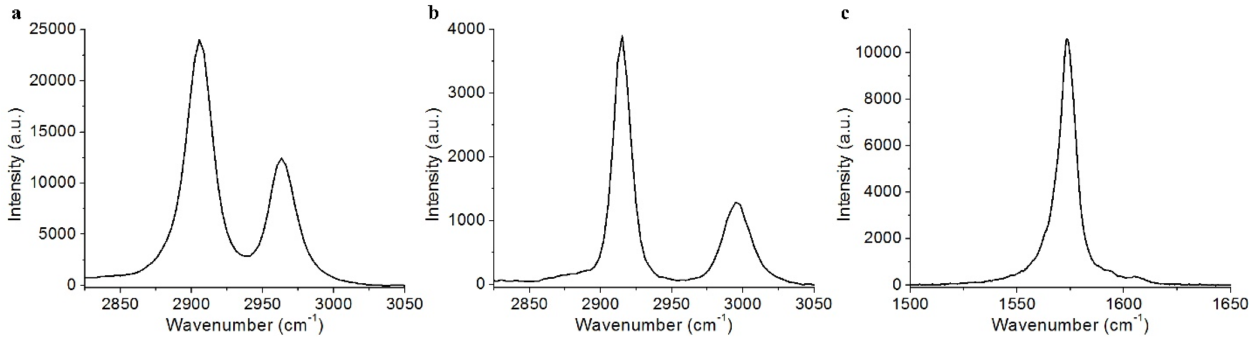

Supplementary Figure 17 | Raman spectra of PDMS, DMSO, and Retinoic acid.

(**a**) The Raman spectrum of PDMS in the C-H vibration region, showing two strong peaks at ~2915 cm^{-1} and ~2965 cm^{-1}. (**b**) The Raman spectrum of DMSO in the C-H vibration region, showing two strong peaks at ~2915 cm^{-1} and 2995 cm^{-1}. (**c**) The Raman spectrum of retinoic acid in the range of 1500-1650 cm^{-1}, showing a strong peak around 1580 cm^{-1}. All the measurements were obtained by a lab-built spontaneous Raman micro-spectroscope.

Supplementary Table 1 Theoretically calculated depth of focus and central lobe radius of the Bessel beams generated by different objectives used in our experiments.

1) Some abbreviations
   M: magnification; **NA**: numerical aperture; **eNA**: effective numerical aperture that means the value really used in our experiments; **FWHM**: full width at half maximum of the longitudinal profile of the generated Bessel beam along the light propagation direction; **SRP**: stimulated Raman scattering.
2) These parameters were calculated at a wavelength of 800 nm.
3) The FWHMs listed in the table were calculated for one Bessel beam. Because of the nonlinear nature of Stimulated Raman scattering, the FWHM of longitudinal profiles of generated SRP signal in light propagation direction would be smaller than the values listed in the table. The FWHMs of SRP signal in longitudinal direction were 2.202, 0.324, 0.204, 0.125 mm for 10X, 25X, 40X, and 60X objectives respectively.
4) The range of the imaged volume was determined by the DOF we can achieve and the scanning limit of our optics. In our current setup, the scanning limit was larger than the achievable DOF. Therefore, the range of the imaged volume was only limited by the FWHM of the SRP signal. Theoretically, the optimal volume we can image should be 2.2×2.2×2.2 mm^3 for 10X objective, 320×320×320 μm^3 for 25X objective, 200×200×200 μm^3 and 125×125×125 μm^3 for 40X and 60X objectives. Such volume can be a bit larger than the optimal value, but it should be smaller than the FWHM of one Bessel beam.
5) In our SRP technology, the central lobe radius determines the theoretical lateral resolution.

Objectives			Depth of Focus	FWHM	Central Lobe Radius
M	NA	eNA	(mm)	(mm)	(μm)
10	0.3	0.19	8.886	3.457	1.55
25	1.05	0.77	1.028	0.508	0.43
40	0.8	0.51	0.646	0.283	0.73
60	1.1	0.88	0.393	0.172	0.34

Supplementary Methods

Materials. Polydimethylsiloxane (PDMS) was purchased from Dow Corning Corp. Dimethyl sulfoxide (DMSO), deuterium oxide (D_2O), and retinoic acid were purchased from Sigma Aldrich. The Raman spectra of PDMS, DMSO, and retinoic acid were measured by a lab-built spontaneous Raman micro-spectroscopy[18]. Each spectrum was acquired for 20 s.

Quartz cuvettes of 2 mm inner width, used in the sensitivity evaluation experiments, were purchased from Precision Cells. Glass capillary tubes used in the SRP tomography, including the square tubes having 100, 200, 400 µm inner widths and the cylindrical tubes having 50, 100, 200 µm inner diameters, were purchased from VitroCom Inc. The low melting point agarose gel (SeaPlaque™ GTG™ agarose) was purchased from Lonza. The square tube having 100 µm inner width and the cylindrical tube having 50 µm inner diameter were used for the SRP tomographic imaging of 10 µm poly(methyl methacrylate) PMMA beads. The square tube with 200 µm inner width and the cylindrical tube with 100 µm inner diameter were used for the SRP tomographic imaging of single adipose cells. The combination of the 400 µm square tube and the 200 µm cylindrical tube was used for the SRP tomographic imaging of the 100 µm polystyrene (PS) bead.

PS and PMMA beads were purchased from Phosphorex Inc. The diameter of beads is 10 µm. The particles were suspended in a 0.1% solution of Tween 20 (Sigma Aldrich) to prevent aggregation. PS beads with a diameter of 100 µm were prepared using the same procedure. For the cell culture, the DMEM medium (high glucose w/L-glutamine) and fetal bovine serum (FBS) were purchased from Thermofisher Scientific. Isobutyllmethylxanthine (IBMX), Dexamethasone (Dex) and Insulin used for adipose cell differentiation were purchased from Sigma Aldrich.

3T3-L1 cell culture and preparation. 3T3-L1 is a mouse derived cell line that is widely used for adipogenesis studies[19]. Under certain simulation conditions, 3T3-L1 cells can differentiate into an adipose phenotype with lipid droplets (LDs) accumulated in the cytosol. In our work, the 3T3-L1 cells were cultured at 37ºC in a humidified atmosphere containing 5% CO_2 and grown continuously in DMEM medium supplemented with 10% FBS, 100 unit/mL penicillin, and 100 µg/mL streptomycin. 1×10^5 cells in 1 mL growth medium were seeded into a petri dish and incubated for 3 to 4 days to encourage adherence and cell confluence. The culture media were changed every 2 days. At 70% cell confluency (day 0), cell differentiation was induced by incubating cells with differentiation medium containing 10% FBS/DMEM, 100× IBMX medium, 1000× Insulin, and 1000× Dex medium. After another 2-day culture (day 2), the medium was changed to the differentiation medium containing 10% FBS/DMEM and 1000× Insulin. At day 4, the medium was changed to 10% FBS/DMEM every two days until day 8. The cells were then detached from the culture dish and suspended in PBS for imaging.

Supplementary references

1. Saar, B. *et al*. Video-rate molecular imaging in vivo with stimulated Raman scattering. *Science* **330**, 1368-1370 (2010).
2. Berto, P., Andresen, E. & Rigneault, H. Background-Free Stimulated Raman Spectroscopy and Microscopy. *Phys. Rev. Lett.* **112**, 053905 (2014).
3. Zhang, D. *et al.* Fast vibrational imaging of single cells and tissues by stimulated Raman scattering microscopy. *Acc. Chem. Res.* **47**, 2282-2290 (2014).
4. Boyd, R. *Nonlinear optics (Third Edition).* (Academic Press, 2008).
5. Zhang, C., Zhang, D. & Cheng, J. Coherent Raman Scattering Microscopy in Biology and Medicine. *Annu. Rev. Biomed. Eng*. **17**, 415-445 (2015).
6. McGloin, D. & Dholakia, K. Bessel beam: diffraction in a new light. *Contemp. Phys.* **46**, 15-28 (2005).
7. Duocastella, M. & Arnold, C. Bessel and annular beams for materials processing. *Laser Photonics Rev*. **6**, 607–621 (2012).
8. Thériault, G. *et al*. Extened two-photon microscopy in live samples with Bessel beams: steadier focus, faster volume scans, and simpler stereoscopic imaging. *Front. Cell. Neurosci.* **8**, 139 (2014).
9. Planchon, T. *et al*. Rapid three-dimensional isotropic imaging of living cells using Bessel beam plane illumination. *Nat. Methods* **8**, 417-423 (2011).
10. Lee, K. & Rolland, J. Bessel beam spectral-domain high-resolution optical coherence tomography with micro-optic axicon providing extended focusing range. *Opt. Lett.* **33**, 1696-1698 (2008).
11. Shi, J. *et al*. Bessel-beam Grueneisen relaxation photoacoustic microscopy with extended depth of field. *J. Biomed. Opt.* **20**, 116002 (2015).
12. Heuke1, S. *et al.* Bessel beam CARS of axially structured samples. *Sci. Rep.* **5**, 10991 (2015).
13. Fahrbach, F., Simon, P. & Rohrbach, A. Microscopy with self-reconstructing beams. *Nat. Photonics* **4**, 780-785 (2010).
14. Herman, R. & Wiggins, T. Rayleigh range and the M^2 factor for Bessel-Gauss beams. *Appl. Opt.* **37**, 3398-3400 (1998).
15. Weber, N. *et al.* Highly compact imaging using Bessel beams generated by ultraminiaturized multi-micro-axcion systems. *J. Opt. Soc. Am. A* **29**, 808-816 (2012).
16. Borghi, R. & Santarsiero, M. M^2 factor of Bessel-Gauss beam. *Opt. Lett*. **22**, 262-264 (1997).
17. Tiwari, S.K. *et al*. Measuring a narrow Bessel beam spod by scanning a charge-coupled device (CCD) pixel. *Meas. Sci. Technol*. **21**, 025038 (2010).
18. Slipchenko, M. *et al*. High-speed vibrational imaging and spectral analysis of lipid bodies by compound Raman microscopy. *J. Phys. Chem. B* **113**, 7681-7686 (2009).
19. Kim, E. *et al.* Recent advances in proteomic studies of adipose tissues and adipocytes. *Int. J. Mol. Sci.* **16**, 4581-4599 (2015).
